# Determination of ETN101, a novel anti-hepatic cancer agent and its two metabolites in rat biological matrices using LC-MS/MS for ADME characterization

**DOI:** 10.3389/fphar.2026.1858527

**Published:** 2026-09-09

**Authors:** Woohyung Jung, Eunbee Jang, Yeoun-Hee Kim, Woojin Kim, Eunbin Kim, Jaewoong Lee, Seongwon Kim, Jae Hun Yoon, Beom Soo Shin, Soyoung Shin, Ju-Hyun Kim, Tae Hwan Kim

**Affiliations:** 1 College of Pharmacy, Daegu Catholic University, Gyeongsan, Republic of Korea; 2 R&D Center, Etnova Therapeutics Corp., Suwon, Republic of Korea; 3 School of Pharmacy, Sungkyunkwan University, Suwon, Republic of Korea; 4 College of Pharmacy, Chung-Ang University, Seoul, Republic of Korea; 5 College of Pharmacy, Yeungnam University, Gyeongsan, Republic of Korea

**Keywords:** ETN101, hepatocellular carcinoma, LC-MS/MS, MBP-11901, pharmacokinetics

## Abstract

ETN101 is a new therapeutic candidate with anti-hepatocellular carcinoma activity. In the present study, three complementary liquid chromatography–tandem mass spectrometry (LC–MS/MS) methods using protein precipitation and matrix-matched calibration were developed for quantifying ETN101 and its metabolites M1 and M2 in rat plasma, tissue homogenates, urine, and feces. The methods were validated with reference to U.S. Food and Drug Administration (FDA) and International Council for Harmonisation (ICH) M10 guidance. A simultaneous assay was used for plasma, urine, and feces, and two LC–MS/MS systems were used for tissue homogenates. Calibration ranges in plasma were 2–2,000 ng/mL for ETN101 and 0.2–2,000 ng/mL for M1 and M2. The corresponding ranges were 50–5,000 and 5–500 ng/mL in tissue homogenates and 20–2,000 and 2–2,000 ng/mL in urine and feces. Across the evaluated matrices, intra- and inter-day accuracy was 87.4%–115.7%, precision was ≤15.0%, and extraction recovery was 56.8%–104.8%. Absolute matrix effects varied among matrices but were reproducible across six individual lots (coefficient of variation ≤15%), and stability met the prespecified criteria. The methods were applied to absorption, distribution, metabolism, and excretion (ADME) studies in rats. After once-daily oral administration at 20 mg/kg for 5 days, ETN101 was slowly absorbed, and M1 and M2 were present in plasma and declined in parallel with the parent drug. No statistically significant fasted–fed differences in 0–24 h systemic exposure were detected on Day 1 or Day 5, and systemic exposure to M2 exceeded that to M1. ETN101 and M2 were quantifiable in all examined tissues (liver, lung, kidney, brain, heart, spleen, testis, thyroid, stomach, small intestine, large intestine, fat, and muscle), whereas M1 was frequently below the limit of quantification in the brain and fat. The highest ETN101 tissue-to-plasma concentration ratios (*K*_*p*_) occurred in the lung and kidney. After intravenous administration at 10 mg/kg, urinary recovery of each analyte was ≤0.03%, and fecal recoveries of ETN101, M1, and M2 were 0.25%, 0.76%, and 2.21%, respectively, giving a low combined recovery (approximately 3.3% of the administered dose). These methods can be selected according to the study purpose and are applicable to further preclinical pharmacokinetic and ADME studies of ETN101.

## 1 Introduction

Hepatocellular carcinoma (HCC) is the most common type of primary liver cancer, accounting for more than 90% of cases. HCC has serious consequences for affected patients given its rapid progression and high rate of metastasis (Park et al., 2022; Asafo-Agyei and Samant, 2020; Dai et al., 2020). HCC is a global health challenge, being the sixth most common cancer in terms of incidence and the fourth leading cause of cancer-related mortality. Its 5-year recurrence rate is 10%–50%, and the 5-year survival rate is approximately 40% (Fares et al., 2020; International Agency for Research on Cancer, 2020). Despite advances in therapeutic strategies, treatment options for advanced HCC remain limited.

For the treatment of HCC, multikinase inhibitors such as sorafenib, lenvatinib, cabozantinib, and regorafenib are used as first- and second-line therapies. However, acquired resistance to sorafenib commonly develops during treatment, and systemic treatment options have expanded to include atezolizumab, sintilimab, camrelizumab, bevacizumab, and pembrolizumab. The first-line treatment of HCC using a combination of atezolizumab (a PD-L1 inhibitor) and bevacizumab (an anti-VEGF antibody) has shown promising results in clinical trials. Although these therapies may produce initial responses, disease progression frequently occurs through primary or acquired drug resistance (Villanueva et al., 2010; Yang et al., 2020; Kudo, 2020; Lee et al., 2020).

ETN101, also known as MBP-11901 (2,3-diamino-N-[4-(2-benzothiazolyl)phenyl]propanamide), is a therapeutic candidate for HCC that has been reported to inhibit VEGFR2-mediated signaling pathways associated with tumor progression and angiogenesis (Park et al., 2022). The molecule consists of a 2-(4-aminophenyl)benzothiazole moiety, a scaffold known to possess antitumor activity (Bradshaw et al., 1998), whose aromatic amine is acylated with a 2,3-diaminopropanoyl group. The presence of two aliphatic primary amines distinguishes ETN101 from its metabolites in terms of polarity, since the benzothiazole moiety itself is lipophilic and poorly soluble in water (Hutchinson et al., 2002). In cell viability assays using various liver cancer cell lines (HepG2, Hep3B, Huh-7, and PLC/PRF/5) and a normal liver cell line, AML12, ETN101 showed potent anti-proliferative effects with IC_50_ values ranging from 5.16 µM in HepG2 cells to 29.41 µM in Huh-7 cells. The anticancer effect of ETN101 was similar to that of sorafenib, but ETN101 showed significantly lower cytotoxicity at low concentrations. Western blot analysis indicated that ETN101 significantly induced apoptosis, whereas sorafenib did not. Following multiple oral administration at doses of 40, 60, and 82 mg/kg for 19 days, tumor growth was significantly inhibited in the HepG2 xenograft tumor model in BALB/c nude mice. In subcutaneous Hep3B and Huh-7 xenograft models, ETN101 treatment significantly decreased tumor volume and weight. No side effects related to drug resistance or rapid tumor growth were observed in the ETN101-treated groups (Park et al., 2022).

A recent *in vitro* study comprehensively characterized the metabolic profile of ETN101 in human, mouse, rat, dog, and monkey hepatocytes (Jung et al., 2024). A total of 34 metabolites were identified, of which M1 (CJM-126, 2-(4-aminophenyl)benzothiazole) is the major metabolite, formed by hydrolysis of the amide bond of ETN101, and M2 (N-acetyl-CJM-126) is formed by subsequent N-acetylation of M1 by NAT1 and NAT2. CYP1A2 was identified as the primary enzyme responsible for the oxidative metabolism of M1. The three analytes differ considerably in basicity and lipophilicity, ranging from the dibasic, comparatively hydrophilic parent drug to the less basic and more lipophilic metabolites M1 and M2, and these differences affect their reversed-phase retention, ionization efficiency, and extraction recovery. In rat hepatocyte incubations, M1 and M2 accounted for approximately 75% and 6% of the total identified metabolite peak area, respectively, whereas each of the remaining metabolites accounted for less than 0.1%. These *in vitro* relative peak areas do not represent absolute metabolite amounts. ETN101 showed similar metabolic stability in human and rat hepatocytes, supporting the use of the rat as a preclinical species for pharmacokinetic evaluation. Accordingly, M1 and M2 were selected as the key metabolites for quantitative monitoring in the present study.

Characterization of the absorption, distribution, metabolism, and excretion (ADME) properties of a drug candidate is essential for understanding its pharmacokinetic behavior and supporting its preclinical and clinical development (Cerny et al., 2020; Zhang et al., 2009). LC–MS/MS-based bioanalytical methods play a central role in this process by enabling the sensitive and selective quantification of parent drugs and their metabolites in biological matrices (Steinmetz and Spack, 2009). However, no bioanalytical method for the determination of ETN101, M1, and M2 in rat biological matrices has been reported to date.

In the present study, we aimed to develop and validate simple and sensitive LC–MS/MS methods for the determination of ETN101, M1, and M2 in rat plasma, tissues, urine, and feces. The validated methods were applied to characterize the pharmacokinetic profiles, tissue distribution, and excretion of ETN101 and its metabolites following oral and intravenous administration in rats.

## 2 Materials and methods

### 2.1 Chemicals and reagents

**FIGURE 1:**
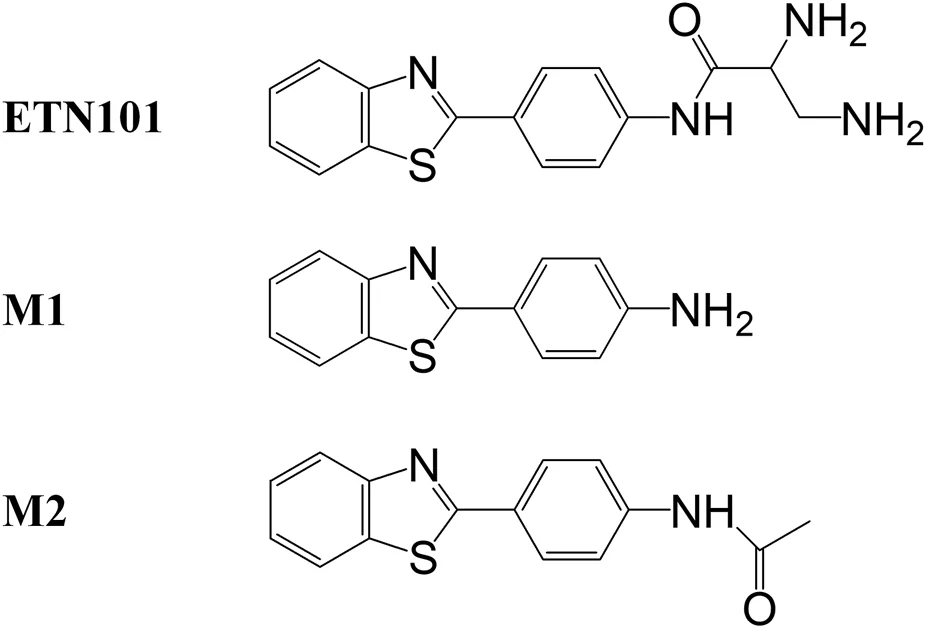
Chemical structures of ETN101, M1, and M2.

ETN101 (C_16_H_16_N_4_OS·2.5HCl·1.5H_2_O) and M2 (N-acetyl-CJM-126, C_15_H_12_N_2_OS) were supplied by Etnova Therapeutics Corp. (Suwon, Korea). M1 (CJM-126, C_13_H_10_N_2_S), amlodipine (internal standard [IS] for plasma, urine, and feces), and glipizide (IS for tissue) were purchased from Sigma-Aldrich Co. (St. Louis, MO, United States). The structures of these chemicals are shown in Figure 1. Acetonitrile, methanol, and water (all HPLC grade) were purchased from J.T. Baker Inc. (Phillipsburg, NJ, United States). Formic acid and ammonium formate were purchased from Merck (Union, NJ, United States).

### 2.2 Instruments and analytical conditions

ETN101, M1, and M2 in rat plasma, urine, and feces (Method A), and ETN101 in tissue samples (Method B) were analyzed using an Agilent 6495 triple-quadrupole mass spectrometer (Agilent Technologies, Santa Clara, CA, United States) coupled to an Agilent 1290 UPLC system (Agilent Technologies, Santa Clara, CA, United States). The compounds were separated on a Poroshell 120 SB-Aq column (2.7 µm, 3.0 × 100 mm; Agilent, Santa Clara, CA, United States) using a gradient elution mobile phase consisting of 0.1% formic acid in 20 mM ammonium formate and 0.1% formic acid in acetonitrile. For Method A, the flow rate was 0.5 mL/min from 0.0 to 10.0 min, increased to 1.0 mL/min from 10.1 to 11.0 min, and returned to 0.5 mL/min from 11.1 to 13.5 min. Method B used a constant flow rate of 0.5 mL/min, and the column oven temperature was maintained at 40 °C.

M1 and M2 in rat tissue samples (Method C) were analyzed using a SCIEX API 4000 triple-quadrupole mass spectrometer (SCIEX, Framingham, MA, United States) coupled to an Agilent 1100 HPLC system (Agilent Technologies, Santa Clara, CA, United States). M1 and M2 were separated on a Luna Omega PS C18 100Å column (1.6 µm, 2.1 × 50 mm) using an isocratic elution consisting of 0.1% formic acid in water and 0.1% formic acid in acetonitrile. The flow rate was 0.25 mL/min, and the column oven temperature was maintained at 40 °C.

The compounds were detected using positive-ion electrospray ionization in multiple reaction monitoring (MRM) mode. On the Agilent 6495, the transitions monitored were m/z 313.1 → 109.0 for ETN101, m/z 227.1 → 118.0 for M1, m/z 269.1 → 227.0 for M2, m/z 409.1 → 238.1 for amlodipine (IS), and m/z 446.2 → 321.1 for glipizide (IS). On the SCIEX API 4000, the transitions monitored were m/z 227.1 → 109.0 for M1, m/z 269.1 → 227.0 for M2, and m/z 446.2 → 321.1 for glipizide (IS). The quantifier product ion of M1 therefore differed between the two instruments, since m/z 118.0 and m/z 109.0 gave the highest signal intensity and the most reproducible response on the Agilent 6495 and the SCIEX API 4000, respectively. A dwell time of 50 ms was applied to each transition. The mass-spectrometric parameters for the two instruments for the quantification of analytes and chromatographic conditions are presented in Tables 1 and 2.

**TABLE 1:** Compound-dependent MS/MS parameters for analytical Methods A–C.

MS/MS system	Analyte	MRM transition (*m/z*)	CE (V)	CAV or CXP (V)^a^
Agilent 6495	ETN101	313.1 → 109.0	58	4
	M1	227.1 → 118.0	38	4
	M2	269.1 → 227.0	50	4
	Amlodipine (IS)	409.1 → 238.1	13	4
	Glipizide (IS)	446.2 → 321.1	17	4
SCIEX API 4000	M1	227.1 → 109.0	45	6
	M2	269.1 → 227.0	35	14
	Glipizide (IS)	446.2 → 321.1	17	20

^a^ All transitions were monitored using positive ESI. CAV was applied on the Agilent 6495 and CXP on the SCIEX API 4000.

ESI, electrospray ionization; IS, internal standard; CE, collision energy; CAV, cell acceleration voltage; CXP, cell exit potential.

**TABLE 2:** LC–MS/MS method assignment and chromatographic conditions for ETN101, M1, and M2 in rat biological matrices.

Parameter	Method A^a^	Method B^a^	Method C^a^
Matrix	Plasma, urine, and fecal homogenate	Tissue homogenate	Tissue homogenate
Analyte(s)	ETN101, M1, and M2	ETN101	M1 and M2
Internal standard	Amlodipine	Glipizide	Glipizide
LC system	Agilent 1290 UPLC	Agilent 1290 UPLC	Agilent 1100 HPLC
MS/MS system	Agilent 6495 triple quadrupole	Agilent 6495 triple quadrupole	SCIEX API 4000 triple quadrupole
Analytical column	Poroshell 120 SB-Aq, 2.7 µm, 3.0 × 100 mm	Poroshell 120 SB-Aq, 2.7 µm, 3.0 × 100 mm	Luna Omega PS C18 100 Å, 1.6 µm, 2.1 × 50 mm (Phenomenex, Torrance, CA, United States)
Column temperature	40 °C	40 °C	40 °C
Mobile phase A	0.1% formic acid in 20 mM ammonium formate	0.1% formic acid in 20 mM ammonium formate	0.1% formic acid in water
Mobile phase B	0.1% formic acid in acetonitrile	0.1% formic acid in acetonitrile	0.1% formic acid in acetonitrile
Elution mode	Gradient^b^	Gradient^c^	Isocratic, A/B = 55:45 (v/v)
Flow rate	0.5–1 mL/min	0.5 mL/min	0.25 mL/min
Injection volume	5 µL	5 µL	10 µL
Run time	13.5 min	7 min	6 min

^a^ Method A, simultaneous determination of ETN101, M1, and M2 in plasma, urine, and fecal homogenate; Method B, determination of ETN101 in tissue homogenates; Method C, simultaneous determination of M1 and M2 in tissue homogenates.

^b^ For Method A, mobile phase A was held at 75% from 0.0 to 5.0 min, decreased to 65% at 6.0 min and held through 10.0 min, decreased to 10% at 10.1 min and held through 11.0 min, and returned to 75% at 11.1 min for re-equilibration through 13.5 min.

^c^ Mobile phase A was held at 80% for 3 min, decreased to 5% over 0.5 min and held for 2 min, followed by re-equilibration to the initial condition for 1.5 min.

### 2.3 Preparation of calibration standards and quality control samples

A stock solution (1 mg/mL, as free base) of ETN101 was prepared by dissolving 137.9 mg of ETN101 (•2.5 HCl •1.5 H_2_O, salt factor: 1.379) in 100 mL of methanol. Stock solutions (1 mg/mL) of M1, M2, and amlodipine were prepared by dissolving 100 mg of M1, M2, and amlodipine in 100 mL of methanol and that of glipizide (500 μg/mL) was prepared by dissolving 50 mg of glipizide in 100 mL methanol. To prepare working solutions, the stock solutions of ETN101, M1, and M2 were diluted with 0.1% formic acid in acetonitrile. To prepare the IS, the amlodipine stock solution was diluted to 500 ng/mL with 0.1% formic acid in acetonitrile and that of glipizide was diluted to 100 ng/mL with methanol as IS.

Plasma calibration samples were prepared by protein precipitation using 0.1% formic acid in acetonitrile. Nominal calibrator concentrations of plasma were 2, 10, 50, 100, 500, 1,000, and 2,000 ng/mL for ETN101 and 0.2, 2, 10, 50, 100, 500, 1,000, and 2,000 ng/mL for M1 and M2. Quality control (QC) samples were prepared by spiking the working solution separately to the blank rat plasma to provide four concentration levels. The QC concentrations of plasma samples were 2 (lower limit of quantification, LLOQ), 6 (low QC), 400 (middle QC), 1,600 (high QC) ng/mL for ETN101 and 0.2 (LLOQ), 0.6 (low QC), 400 (middle QC), 1,600 (high QC) ng/mL for M1 and M2.

Tissue-homogenate calibration samples were prepared by double protein precipitation with methanol. Nominal calibrator concentrations in tissue homogenates were 50, 100, 200, 500, 1,000, 2,000, and 5,000 ng/mL for ETN101 and 5, 10, 20, 50, 100, 200, and 500 ng/mL for M1 and M2. QC samples were prepared by separately spiking the working solutions into blank rat tissue homogenate. The tissue-homogenate QC concentrations were 50 (LLOQ), 150 (low QC), 800 (middle QC), and 4,000 (high QC) ng/mL for ETN101 and 5 (LLOQ), 15 (low QC), 80 (middle QC), and 400 (high QC) ng/mL for M1 and M2.

Urine and feces calibration samples were prepared by double protein precipitation with methanol. Nominal calibrator concentrations of urine and feces were 20, 50, 100, 200, 500, 1,000, and 2,000 ng/mL for ETN101 and 2, 20, 50, 100, 200, 500, 1,000, and 2,000 ng/mL for M1 and M2. QC samples were prepared by spiking the working solution separately to the blank rat urine and feces. The QC concentrations of urine and feces samples were 20 (LLOQ), 60 (low QC), 400 (middle QC), 1,600 (high QC) ng/mL for ETN101 and 2 (LLOQ), 6 (low QC), 400 (middle QC), 1,600 (high QC) ng/mL for M1 and M2.

Stock and working solutions were stored at −20 °C. QC samples were stored at −80 °C until analysis.

### 2.4 Sample preparation

Standard plasma samples were prepared by spiking 50 µL of blank rat plasma with 5 µL of the working solution. To each plasma standard sample, the IS (amlodipine, 500 ng/mL, 50 µL) and 0.1% formic acid in acetonitrile (95 µL) were added and mixed on a vortex mixer for 1 min. The mixture was centrifuged for 10 min at 15,000 × *g* for protein precipitation. The supernatant (70 µL) was reconstituted with 0.1% formic acid in 20 mM ammonium formate in water (30 µL). A portion (5 µL) was injected into LC–MS/MS.

Tissue samples were weighed and homogenized in water at a concentration of 0.5 g/mL. Water was used rather than an isotonic buffer such as phosphate-buffered saline or normal saline to avoid the introduction of non-volatile salts, which are a known source of ion suppression in electrospray ionization (King et al., 2000). Standard tissue samples were prepared by spiking 50 µL of blank rat tissue homogenate with 5 µL of the working solution. To each tissue standard sample, the IS (glipizide, 100 ng/mL, 50 µL) and methanol (95 µL) were added and mixed on a vortex mixer for 1 min. The mixture was centrifuged for 10 min at 15,000 × *g* for protein precipitation. Methanol (150 µL) was added to the supernatant (150 µL) for double protein precipitation, and the mixture was mixed on a vortex mixer for 1 min. The mixture was centrifuged for 10 min at 15,000 × *g*, and the supernatant (180 µL) was reconstituted with water (20 µL). A portion of 5 µL for Method B or 10 µL for Method C was injected into LC–MS/MS.

Urine samples were filtered with a PTFE syringe filter, and feces samples were weighed and homogenized in methanol at 0.25 g/mL. Standard urine and feces samples were prepared by spiking 100 µL of blank rat urine or fecal homogenate with 100 µL of the working solution. To each of the urine and feces standard samples, the IS (amlodipine, 500 ng/mL, 100 µL) and methanol (700 µL) were added and mixed on a vortex mixer for 1 min. The mixture was centrifuged for 10 min at 15,000 × *g* for protein precipitation. Methanol (150 µL) was added to the supernatant (150 µL) for double protein precipitation, and the mixture was mixed on a vortex mixer for 1 min. The mixture was centrifuged for 10 min at 15,000 × *g*, and the supernatant (200 µL) was reconstituted with 0.1% formic acid in 20 mM ammonium formate in water (200 µL). A portion (5 µL) was injected into LC–MS/MS.

Quality control and study samples were processed in the same manner as the calibration standards, with the working solution replaced by an equal volume of the corresponding blank solvent so that the matrix and final solvent volumes were identical.

### 2.5 Method validation

#### 2.5.1 Selectivity, linearity, and sensitivity

Selectivity was evaluated by comparing the chromatograms of blank matrices from six rats with blank matrices spiked with ETN101, metabolites, and IS in the appropriate rat matrices. Linearity was evaluated from calibration curves. Calibration curves were calculated with the weighted linear regression (1/*x* ^2^) method using the peak area ratio of analytes *versus* nominal concentrations to the IS. The calibration curves for analyzing biological matrices were evaluated in accordance with FDA and ICH guidelines (U.S. Food and Drug Administration, 2018; International Council for Harmonisation, 2022). Correlation coefficients greater than 0.99 were considered acceptable. LLOQ was used to evaluate sensitivity through a signal-to-noise ratio (S/N ratio). An S/N ratio greater than 10 was considered acceptable. Carryover was assessed by injecting a blank matrix sample immediately after the upper limit of quantification (ULOQ) sample. The response in the analyte and IS retention-time windows did not exceed 20% of the LLOQ and 5% of the IS response, respectively.

#### 2.5.2 Accuracy and precision

Intra-day accuracy and precision were evaluated using five replicates at each QC level within a single day. Inter-day accuracy and precision were assessed by analyzing the QC samples over five consecutive days. Precision was expressed as the coefficient of variation of each concentration, and the accuracy was expressed as the percentage of mean measured *versus* nominal concentrations. An accuracy of 85%–115% (LLOQ: 80%–120%) and a precision of ≤15% (LLOQ: ≤20%) were accepted in this validation assay.

#### 2.5.3 Stability

The stability of ETN101, M1, and M2 was evaluated under four conditions using five replicates in plasma and three replicates in the other matrices. Rat plasma, tissue, urine, and feces were spiked with ETN101, M1, and M2 at the LQC and HQC concentrations, and the spiked samples were kept at 4 °C for 2 h for short-term stability, stored at −80 °C for 5 weeks for long-term stability, or subjected to three freeze-thaw cycles for freeze-thaw stability. Processed sample stability was assessed by keeping the extracted samples in the autosampler at 4 °C for 24 h prior to injection.

#### 2.5.4 Matrix effect, extraction recovery, and process efficiency

The matrix effect, extraction recovery, and process efficiency were evaluated in four representative matrices (plasma, liver, urine, and feces) at the LQC and HQC levels, each prepared from six individual lots (n = 6). For each analyte, three sets of samples were prepared: neat standard solutions in the extraction solvent (set A), blank matrices spiked with the analytes after extraction (set B), and blank matrices spiked with the analytes before extraction (set C) (Matuszewski et al., 2003). Following the post-extraction addition approach, all three parameters were derived from the raw analyte peak areas: the matrix effect as the ratio of set B to set A, the extraction recovery as the ratio of set C to set B, and the process efficiency as the ratio of set C to set A, each expressed as a percentage. The internal standard was added before extraction in all sets, consistent with the routine sample analysis, and the parameters were therefore calculated without internal standard normalization.

### 2.6 Pharmacokinetics, tissue distribution, and excretion studies

The pharmacokinetic study and tissue distribution were evaluated following oral administration, and the excretion study was evaluated in rats following intravenous injection of ETN101. Male Sprague-Dawley rats (8–10 weeks, body weight, 240–290 g; Hyochang Science, Daegu, Korea) were kept in plastic cages with free access to standard rat diet and water. The rats were maintained at a temperature of 23 °C ± 2 °C with a 12 h light/12 h dark cycle, with lights on from 10 a.m. to 10 p.m. with 150–300 Lux and relative humidity of 50% ± 10% and were acclimatized for at least 1 week prior to the experiment. For the pharmacokinetic study, rats were cannulated using polyethylene tubing (0.58 mm i.d., 0.96 mm o.d., Natsume, Tokyo, Japan) in the jugular vein after intraperitoneal injection of Zoletil 50 (Virbac, Carros, France) at a dose of 0.9 mL/kg. All procedures were approved by the Institutional Animal Care and Use Committee (IACUC) at Daegu Catholic University and conducted in accordance with the institutional guidelines for the care and use of laboratory animals (Approval No. DCU-2021-010).

All ETN101 dose levels in the *in vivo* studies are expressed as free-base equivalents. After a 1-day recovery period, rats were assigned to fasted and fed groups. In the fasted group, rats were deprived of food for approximately 12 h before each daily administration and were provided with a defined amount of food 4–6 h after dosing. In the fed group, rats had free access to food throughout the study period. Rats received 1 mL/kg of water orally immediately prior to each ETN101 dose. ETN101 dissolved in normal saline (20 mg/mL) was orally administered once daily for five consecutive days at 20 mg/kg. Serial venous blood samples were collected *via* the jugular vein at 0 (pre-dose), 0.25, 0.5, 0.75, 1, 2, 4, 6, 8, 12, and 24 h after the first dose on Day 1 and after the fifth dose on Day 5. The Day 1 and Day 5 pharmacokinetic analyses each included three individual profiles per group. Additional pre-dose samples were collected immediately before dosing on Days 3, 4, and 5, corresponding to study times of 48, 72, and 96 h, respectively. Plasma was harvested by centrifugation at 15,000 × *g* for 10 min and stored at −80 °C until analysis.

At 24 h after the fifth dose on Day 5, rats in both the fasted and fed groups (n = 3 per group) were sacrificed under anesthesia, and the systemic circulation was perfused with physiological saline *via* the abdominal aorta to remove residual blood from the tissues. The liver, lung, kidney, brain, heart, spleen, testis, thyroid, stomach, small intestine, large intestine, fat, and muscle were collected, blotted dry, weighed, and stored at −80 °C until analysis.

For the excretion study, a separate group of rats (n = 6) received a single intravenous administration of ETN101 at 10 mg/kg in normal saline. Rats were housed individually in stainless-steel metabolic cages, and urine and feces samples were collected separately at time intervals of 0–24, 24–48, 48–72, 72–96, and 96–144 h after administration and stored at −80 °C until analysis.

### 2.7 Data analysis

Plasma concentration-time data were analyzed by non-compartmental analysis using Phoenix™ WinNonlin® 8.3.4 (Certara USA, Inc., Princeton, NJ, United States). Parameters were calculated separately for the 0–24 h interval after the first dose on Day 1 and the fifth dose on Day 5. The parameters included the apparent terminal half-life (t_1/2_), the maximum observed plasma concentration (C_max_), the time to C_max_ (T_max_), and the area under the plasma concentration-time curve over the 24-h dosing interval following the Day 1 and Day 5 doses (AUC_0–24,Day 1_ and AUC_0–24,Day 5_, respectively). AUC_0–24_ was calculated using the trapezoidal method. If an individual parameter could not be estimated reliably, it was treated as missing and the summary was based on the available individual estimates. The area under the concentration-time curve extrapolated to infinity (AUC_inf_) was not estimated, as sampling ended at 24 h after each dose. For each animal, the tissue-to-plasma concentration ratio (*K*_*p*_) was calculated by dividing the tissue concentration by the plasma concentration obtained from the same animal at the same sampling time. The individual ratios were summarized as mean ± SD. The cumulative recovery (%) was calculated as the total amount of analyte recovered in urine or feces divided by the administered ETN101 dose. For M1 and M2, recovered amounts were converted to molar equivalents of ETN101 using their respective molecular weights and expressed as a percentage of the administered ETN101 molar dose.

Statistical analyses were performed using IBM SPSS Statistics® 21 (IBM Corp., Armonk, NY, United States). Given the small sample size, all comparisons were considered exploratory. The t_1/2_, C_max_, and AUC_0–24_ values were compared between the fasted and fed groups on each day using unpaired two-sample t-tests. T_max_ was summarized descriptively as median (range). Tissue concentrations and *K*_*p*_ values were compared between the fasted and fed groups for each analyte and tissue using exploratory unpaired two-sample t-tests without adjustment for multiple comparisons. A two-sided p value <0.05 was considered statistically significant.

## 3 Results

### 3.1 Development and validation of an LC-MS/MS method

**FIGURE 2:**
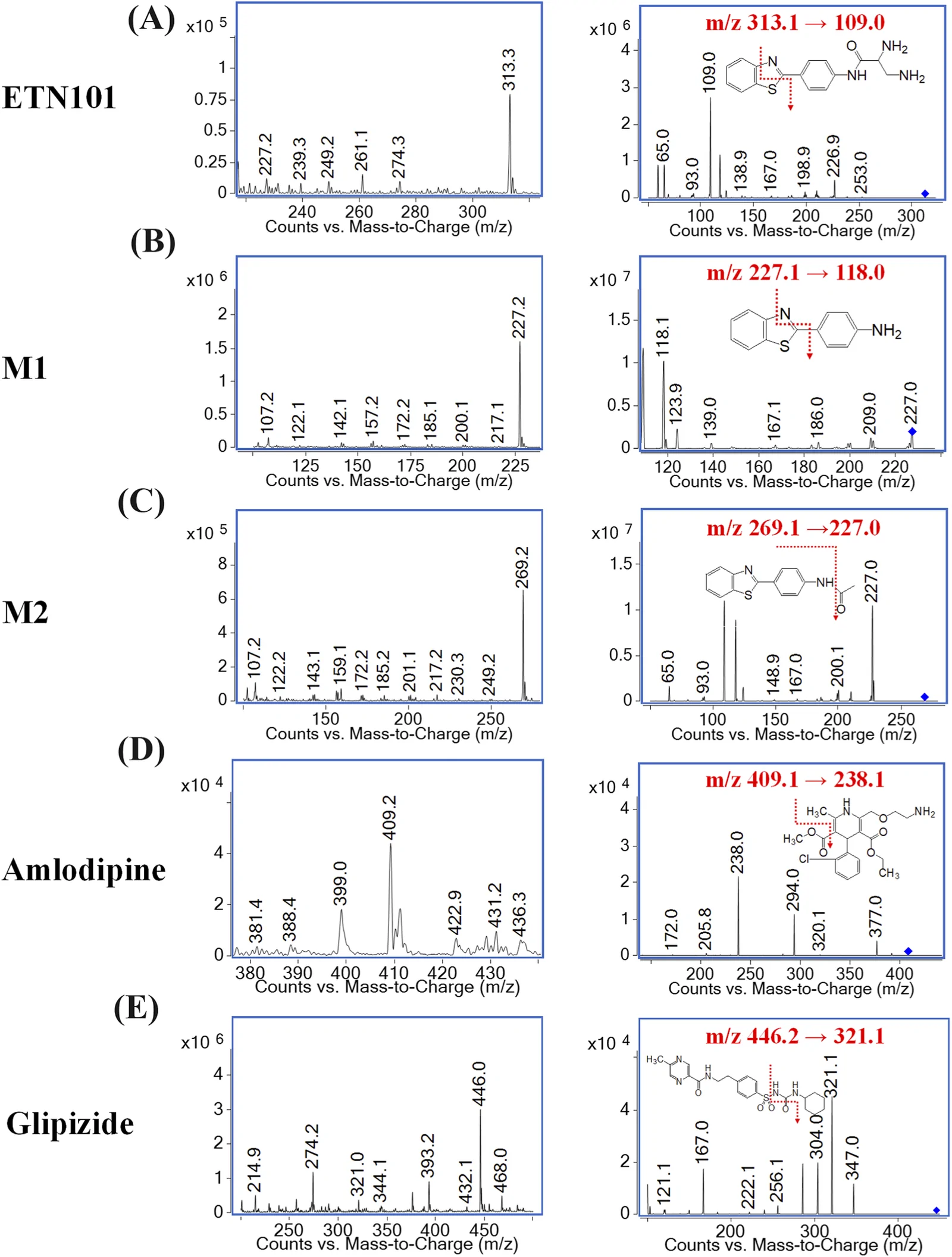
Positive Q1 (left) and product ion mass spectra (right) of protonated **(A)** ETN101, **(B)** M1, **(C)** M2, and **(D and E)** IS (amlodipine, glipizide) obtained with the Agilent 6495 mass spectrometer.

In the positive Q1 mass scan, the protonated analytes [M + H]^+^ of ETN101, M1, M2, and IS were observed at m/z 313.1, 227.1, 269.1, 409.1 (amlodipine), and 446.2 (glipizide), respectively. In the product ion scans obtained on the Agilent 6495, the MRM transitions selected for quantification were m/z 313.1 → 109.0 for ETN101, m/z 227.1 → 118.0 for M1, m/z 269.1 → 227.0 for M2, m/z 409.1 → 238.1 for amlodipine, and m/z 446.2 → 321.1 for glipizide (Figure 2). On the SCIEX API 4000, which was used for the determination of M1 and M2 in tissue homogenates, the transitions were m/z 227.1 → 109.0 for M1, m/z 269.1 → 227.0 for M2, and m/z 446.2 → 321.1 for glipizide (Supplementary Figure S1). The quantifier product ion of M1 differed between the two instruments, as m/z 118.0 and m/z 109.0 provided the highest signal intensity and the most reproducible response on the Agilent 6495 and the SCIEX API 4000, respectively (Table 1).

**FIGURE 3:**
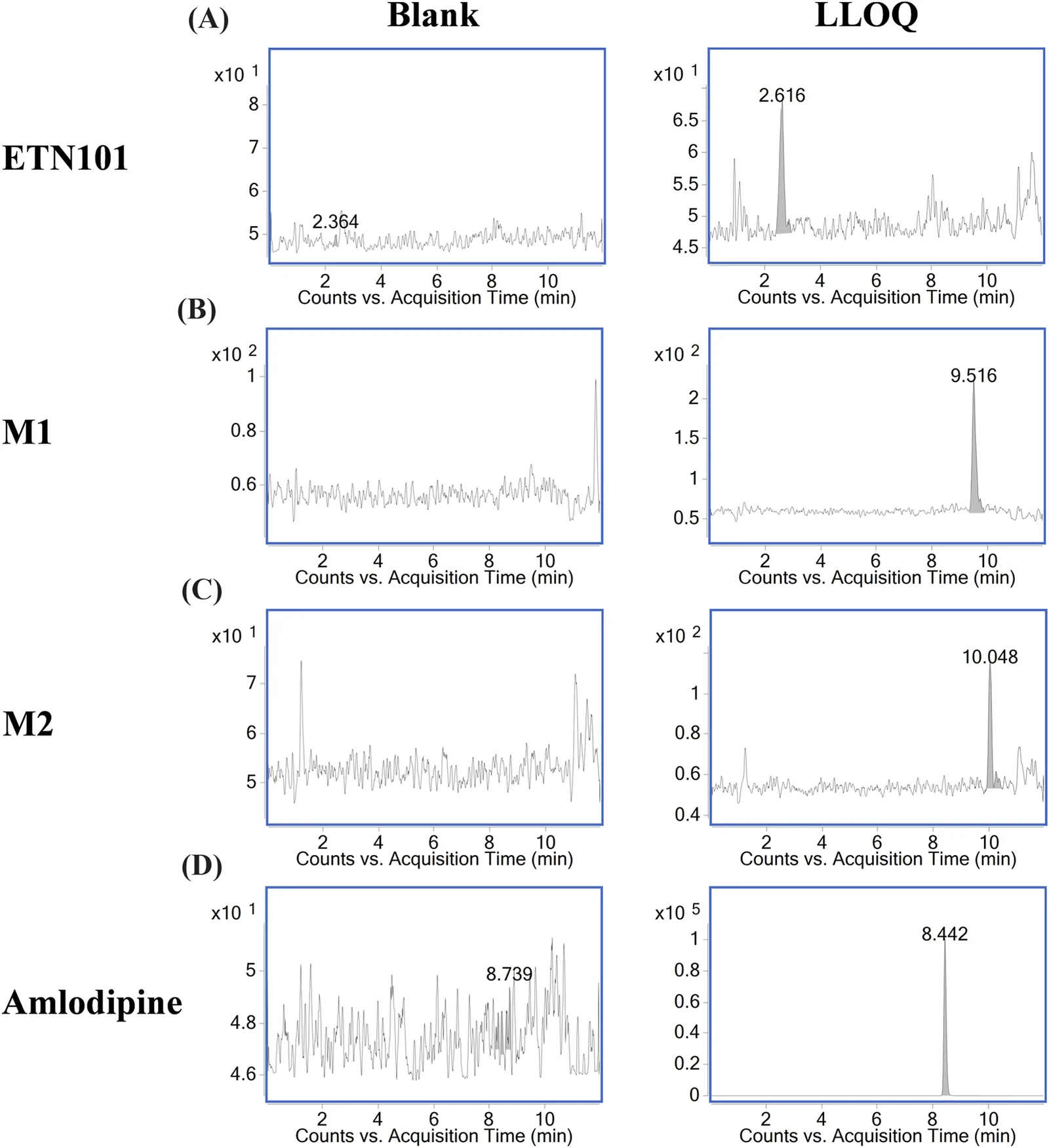
Representative MRM chromatograms of **(A)** ETN101, **(B)** M1, **(C)** M2, and **(D)** IS in blank plasma (left), plasma spiked with LLOQ (right).

Six blank rat plasma samples from different rats were investigated to assess the selectivity of the method. The analytes in rat plasma were separated from endogenous interferences, and the S/N ratios were >10 under the established chromatographic conditions (Figure 3). The method was linear over the range of 2–2,000 ng/mL for ETN101 and 0.2–2,000 ng/mL for M1 and M2. Calibration curves were fitted by linear regression using a weighting of 1/x^2^ to improve the accuracy at low concentrations. The correlation coefficients were above 0.99 (Supplementary Figure S2).

Intra- and inter-day accuracy and precision were assessed at the LLOQ, LQC, MQC, and HQC on five different days. The intra-day accuracy was 87.42%–115.66% for ETN101, 97.26%–109.68% for M1, and 97.46%–108.54% for M2. The inter-day accuracy was 95.68%–99.70% for ETN101, 90.38%–104.10% for M1, and 88.56%–103.92% for M2. The intra- and inter-day precisions were ≤8.98% and ≤10.76%, respectively (Table 3). Short-term, freeze-thaw, processed sample, and long-term stabilities were determined for ETN101, M1, and M2 in rat plasma. The percentage of the calculated *versus* nominal concentration ratio at HQC and LQC was 91.56%–110.62% for ETN101, 94.26%–109.90% for M1, and 88.74%–109.40% for M2 (Table 4).

**TABLE 3:** Intra- and inter-day accuracy and precision for ETN101, M1, and M2 in rat plasma (n = 5).

Analyte	QC concentration (ng/mL)	Intra-day (n = 5)		Inter-day (n = 5)	
		Accuracy (%)	Precision (%CV)	Accuracy (%)	Precision (%CV)
ETN101	2	115.66	3.33	99.22	10.76
	6	95.2	5.91	95.68	6.65
	400	88.18	3.33	99.58	5.09
	1,600	87.42	2.38	99.7	1.53
M1	0.2	109.68	4.27	104.1	9.25
	0.6	105.56	4.71	90.38	4.13
	400	97.26	4.04	99.2	3.44
	1,600	101.16	2.18	96.68	0.75
M2	0.2	99.44	8.98	101.58	6.59
	0.6	97.46	4.55	88.56	3.18
	400	97.7	4.94	98.34	3.47
	1,600	108.54	2.46	103.92	1.26

**TABLE 4:** Stability of ETN101, M1, and M2 in rat plasma at low and high QC concentrations after short-term storage (4 °C, 2 h), three freeze-thaw cycles, processed sample storage (4 °C, 24 h), and long-term storage (−80 °C, 5 weeks) (n = 5).

Analyte	QC level	Short-term (%)		Freeze-thaw (%)		Processed sample (%)		Long-term (%)	
		Accuracy (%)	Precision (%CV)	Accuracy (%)	Precision (%CV)	Accuracy (%)	Precision (%CV)	Accuracy (%)	Precision (%CV)
ETN101	LQC	104.74	4.49	104.74	4.84	105.06	2.12	98.76	4.80
	HQC	110.62	3.91	106.88	0.91	91.56	2.35	107.06	4.44
M1	LQC	100.76	4.37	97.90	1.63	107.06	2.13	105.08	5.31
	HQC	97.08	2.58	94.26	0.63	95.04	1.86	109.90	0.39
M2	LQC	95.32	1.61	88.74	4.98	93.64	3.50	103.48	2.69
	HQC	97.76	1.22	98.34	1.23	105.94	1.67	109.40	1.27

**FIGURE 4:**
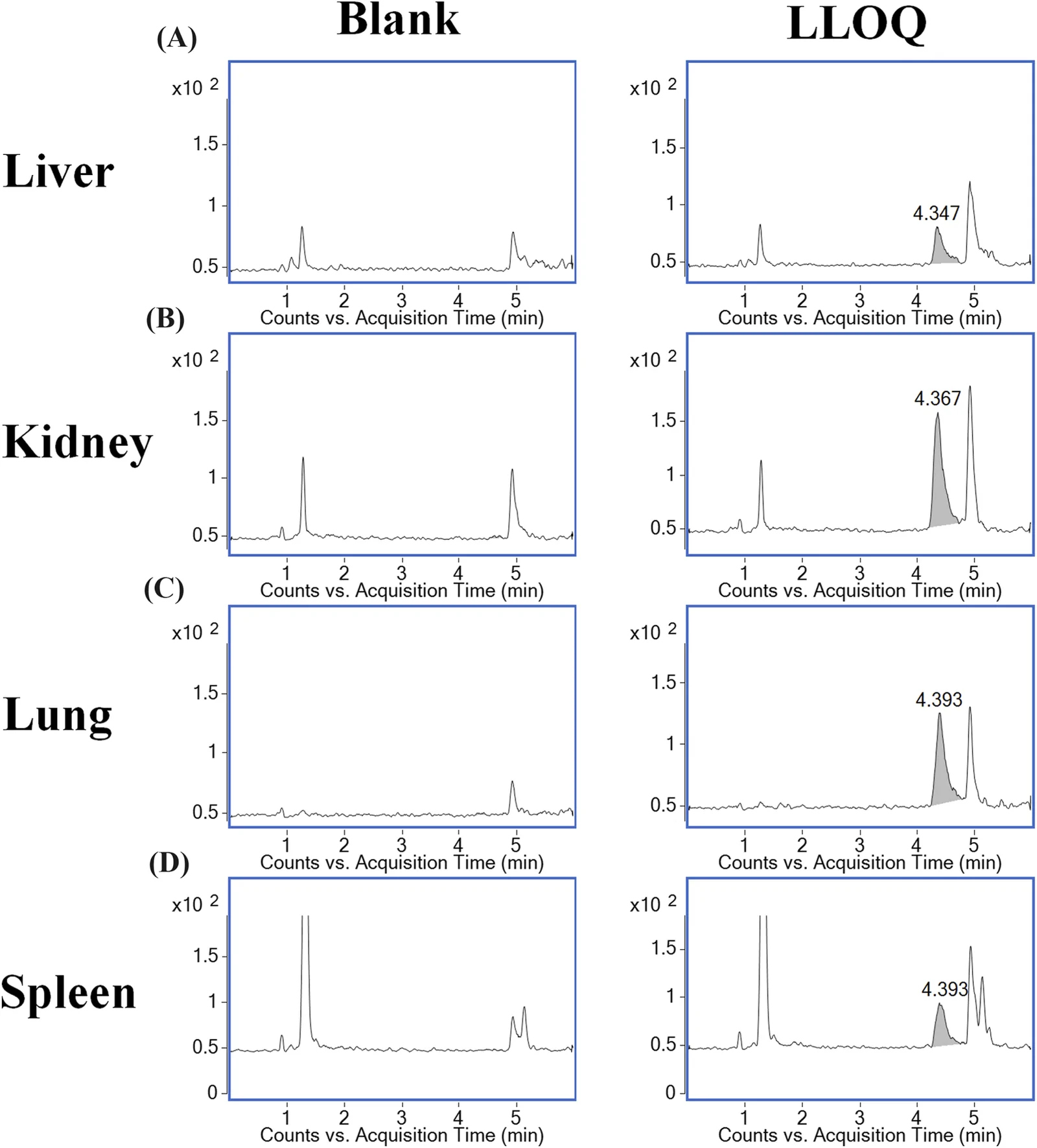
Representative MRM chromatograms of ETN101 in blank **(A)** liver, **(B)** kidney, **(C)** lung, and **(D)** spleen (left) and the corresponding tissues spiked with LLOQ (right).

**FIGURE 5:**
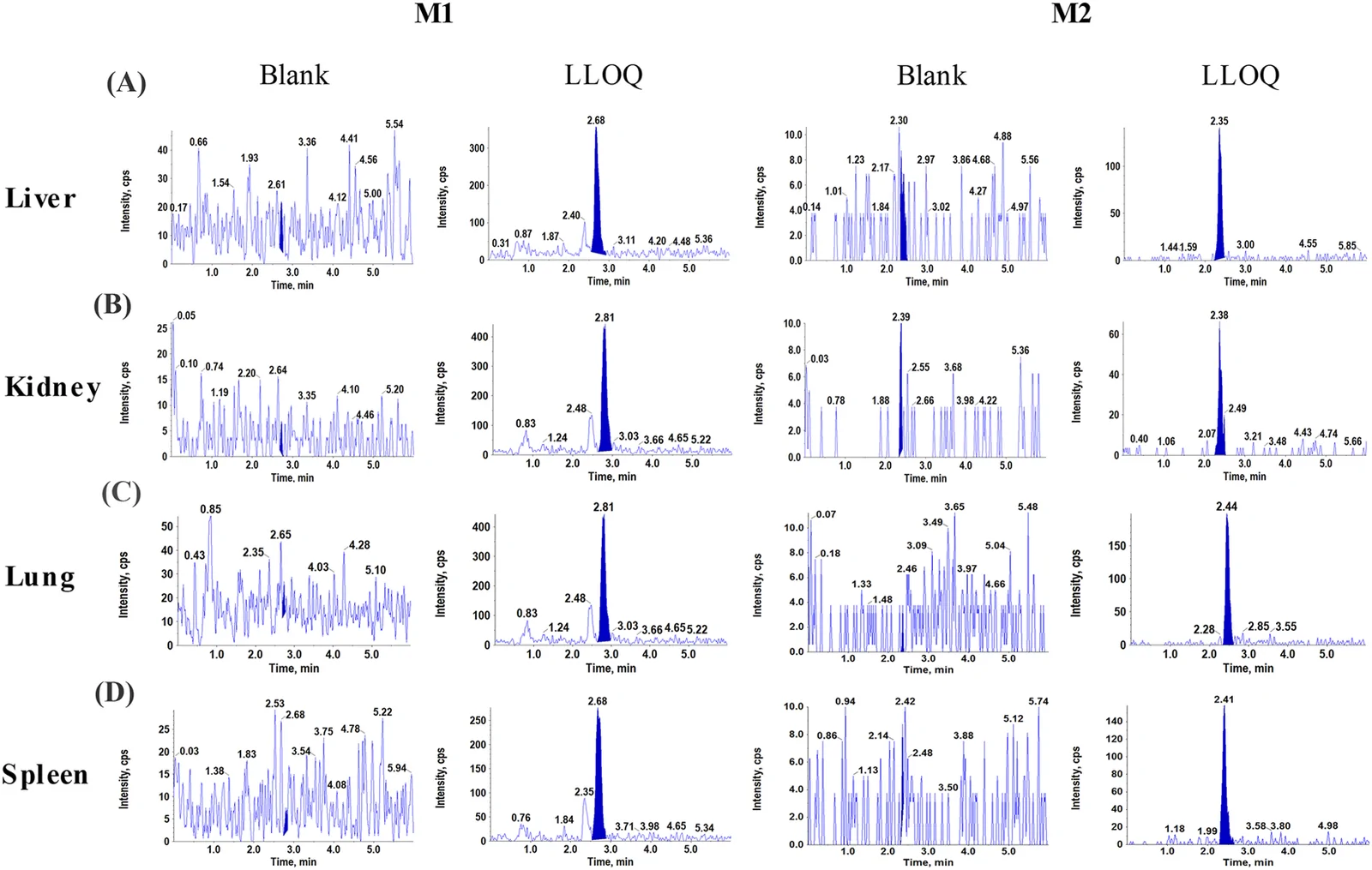
Representative MRM chromatograms of M1, M2 in blank **(A)** liver, **(B)** kidney, **(C)** lung, and **(D)** spleen (left) and the corresponding tissues spiked with LLOQ (right).

Six blank rat biological matrices (tissue, urine, and feces) from different rats were investigated. The analytes were separated from endogenous interferences, and the S/N ratios were >10 (Figures 4, 5; Supplementary Figure S3). The method was linear over 50–5,000 ng/mL for ETN101 and 5–500 ng/mL for M1 and M2 in tissues, and over 20–2,000 ng/mL for ETN101 and 2–2,000 ng/mL for M1 and M2 in urine and feces. The correlation coefficients were above 0.99 (Supplementary Figures S4–S6). The stabilities in rat biological matrices were within 85.33%–113.27% for ETN101, 86.30%–112.00% for M1, and 88.27%–113.00% for M2 (Supplementary Table S2). The matrix effect ranged from 47.32% to 160.21% for ETN101, from 68.52% to 103.51% for M1, and from 83.89% to 121.03% for M2. The extraction recovery ranged from 56.83% to 93.10% for ETN101, from 73.27% to 100.43% for M1, and from 74.88% to 104.78% for M2. The process efficiency ranged from 31.74% to 102.14% for ETN101, from 68.65% to 103.81% for M1, and from 81.69% to 105.44% for M2. Ion suppression was most pronounced for ETN101 in liver, whereas ion enhancement was observed in feces. The evaluated accuracy, precision, and stability results met the prespecified acceptance criteria in each matrix (Supplementary Tables S1 and S2). For the four representative matrices evaluated at LQC and HQC, the between-lot CVs of the absolute matrix effect, extraction recovery, and process efficiency were ≤15% for all analytes (Supplementary Tables S3–S5).

### 3.2 Pharmacokinetic study following repeated oral administration of ETN101

**FIGURE 6:**
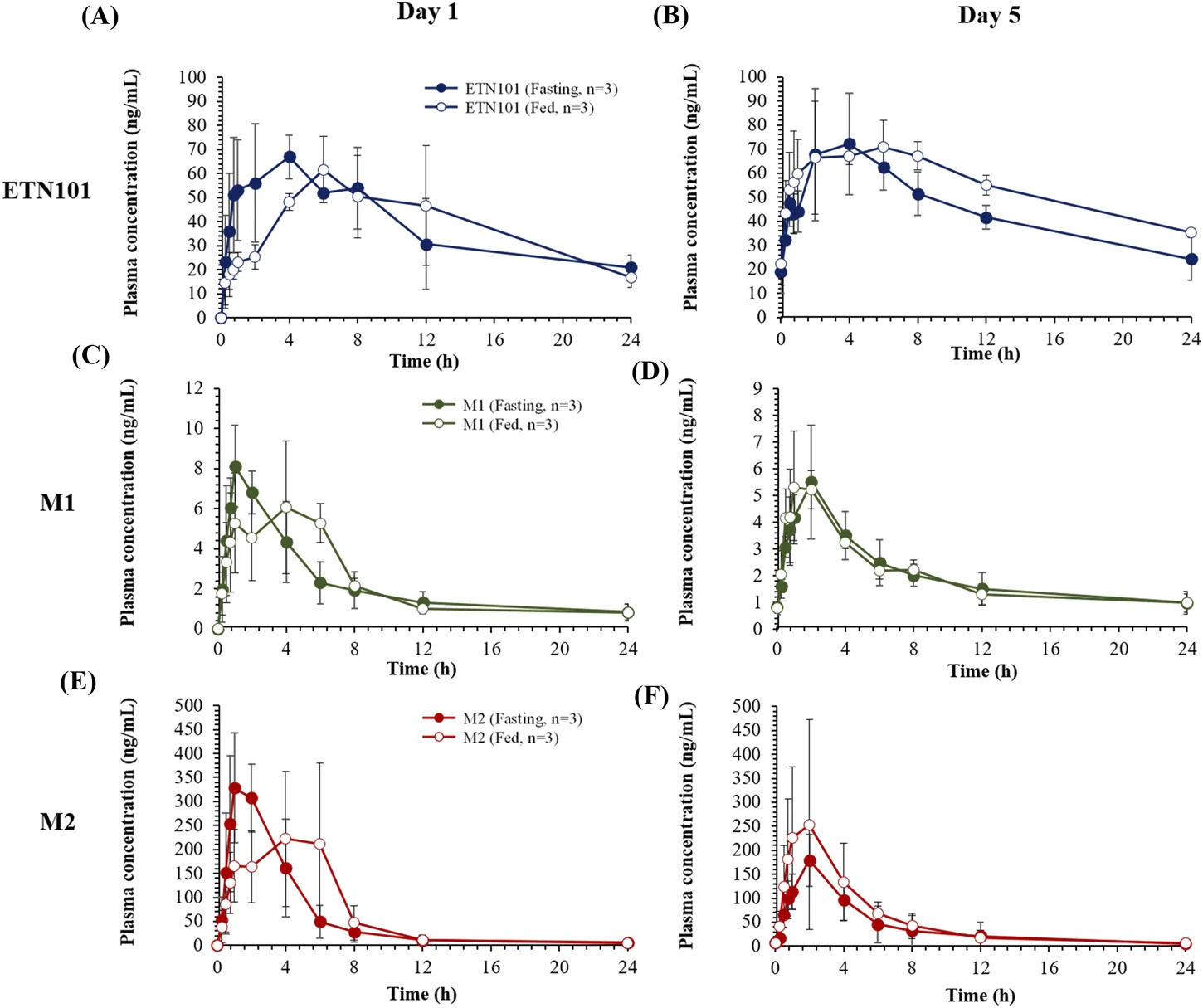
Mean plasma concentration-time profiles of **(A,B)** ETN101, **(C, D)** M1, and **(E,F)** M2 during 0‐24 h after the first oral dose on Day 1 **(A,C,E)** and during the 24‐h dosing interval after the fifth oral dose on Day 5 **(B,D,F)** in fasted and fed rats administered ETN101 at 20 mg/kg once daily (n = 3 per group, arithmetic mean ± SD, linear scale). For the Day 5 profiles, time zero represents the pre‐dose concentration immediately before the fifth dose.

The developed assay was applied to a repeated-dose pharmacokinetic study of ETN101 at 20 mg/kg once daily for five consecutive days. Day 1 and Day 5 plasma concentration-time profiles are compared in Figure 6, and the complete 0–120 h profiles and pre-dose concentrations are presented in Supplementary Figure S7 and Supplementary Table S6, respectively. Pharmacokinetic parameters are summarized in Table 5 as geometric mean (GSD), except T_max_, which is presented as median (range).

**TABLE 5:** Pharmacokinetic parameters of ETN101, M1, and M2 during the 24-h dosing interval after the first dose on Day 1 and the fifth dose on Day 5 following oral administration of ETN101 at 20 mg/kg once daily to fasted and fed rats (n = 3 per group).

Analyte	Parameter	Fasted		Fed	
		Day 1	Day 5	Day 1	Day 5
ETN101	t_1/2_ (h)	11.72 (1.64)	13.11 (1.3)	10.32 (1.35)	12.42 (1.35)
	T_max_ (h)	8 (2–12)	4 (2–6)	6 (4–12)	6 (2–6)
	C_max_ (ng/mL)	67.13 (1.23)	76.48 (1.27)	61.96 (1.23)	80.85 (1.09)
	AUC_0–24_ (ng·h/mL)	941.4 (1.07)	1,058.42 (1.14)	880.32 (1.38)	1,302.49 (1.11)
M1	t_1/2_ (h)	12.73 (1.85)	13.51 (1.05)	12.18 (1.66)	17.30 (1.55)
	T_max_ (h)	2 (1–4)	2 (2–4)	4 (1–12)	2 (1–2)
	C_max_ (ng/mL)	6.87 (1.5)	5.46 (1.4)	6.18 (1.41)	5.17 (1.17)
	AUC_0–24_ (ng·h/mL)	43.79 (1.26)	48.80 (1.2)	45.83 (1.07)	46.66 (1.18)
M2	t_1/2_ (h)	8.63 (3.2)	9.10 (2.2)	10.48 (2.32)	7.40 (1.21)
	T_max_ (h)	2 (1–4)	2 (2–4)	4 (1–6)	2 (1–2)
	C_max_ (ng/mL)	230.07 (2.04)	185.03 (1.25)	240.94 (1.63)	162.67 (2.74)
	AUC_0–24_ (ng·h/mL)	925.95 (1.5)	908.83 (1.5)	1,367.31 (1.85)	957.98 (2.29)

Data are presented as geometric mean (geometric standard deviation), except T_max_, which is presented as median (range).

ETN101 showed relatively late and variable observed T_max_ values on Day 1 in both the fasted and fed groups. The concentration-time profiles of M1 and M2 paralleled that of ETN101, and their observed T_max_ values were generally earlier than that of the parent drug. On Day 5, the profiles of all three analytes were broader and more sustained than those observed after the first dose.

For ETN101, the geometric mean C_max_ and AUC_0–24,Day 1_ were 67.13 ng/mL and 941.40 ng·h/mL in the fasted group and 61.96 ng/mL and 880.32 ng·h/mL in the fed group. On Day 5, the corresponding C_max_ and AUC_0–24,Day 5_ values were 76.48 ng/mL and 1,058.42 ng·h/mL in the fasted group and 80.85 ng/mL and 1,302.49 ng·h/mL in the fed group. The apparent terminal half-life was approximately 10–13 h across the ETN101 groups.

For M1, the geometric mean C_max_ and AUC_0–24_ were 6.87 ng/mL and 43.79 ng·h/mL (fasted) and 6.18 ng/mL and 45.83 ng·h/mL (fed) on Day 1, and 5.46 ng/mL and 48.8 ng·h/mL (fasted) and 5.17 ng/mL and 46.66 ng·h/mL (fed) on Day 5. For M2, the corresponding values were 230.07 ng/mL and 925.95 ng·h/mL (fasted) and 240.94 ng/mL and 1,367.31 ng·h/mL (fed) on Day 1, and 185.03 ng/mL and 908.83 ng·h/mL (fasted) and 162.67 ng/mL and 957.98 ng·h/mL (fed) on Day 5.

No statistically significant differences in t_1/2_, C_max_, or AUC_0–24_ were detected between the fasted and fed groups for any analyte on either day (p > 0.05). Given the small sample size (n = 3 per group), these findings should be interpreted as an absence of a detected difference rather than evidence of pharmacokinetic equivalence or absence of a food effect.

In a descriptive Day 1 *versus* Day 5 comparison, ETN101 AUC_0–24_ was numerically higher on Day 5 in both the fasted and fed groups, M1 AUC_0–24_ changed little, and M2 AUC_0–24_ did not show a consistent increase across the two groups. Given that only three animals were evaluated per group, these observations do not establish the presence or absence of drug accumulation.

### 3.3 Tissue distribution following repeated oral administration of ETN101

**FIGURE 7:**
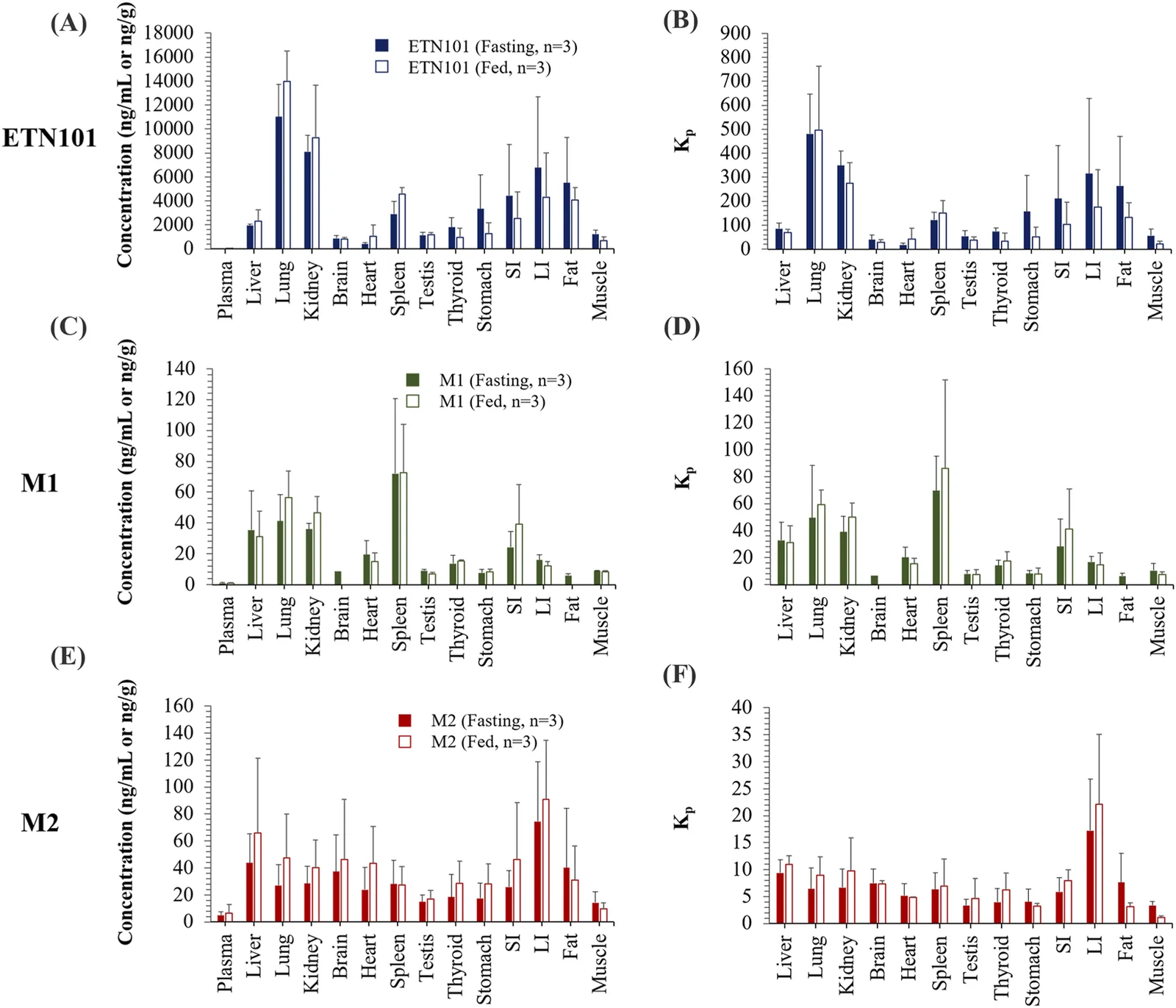
Plasma and tissue concentrations of **(A)** ETN101, **(C)** M1, and **(E)** M2 and tissue-to-plasma concentration ratios (Kp) of **(B)** ETN101, **(D)** M1, and **(F)** M2 at 24 h after the fifth oral dose on Day 5 in fasted and fed rats administered ETN101 at 20 mg/kg once daily (n = 3 per group, mean ± SD). No statistically significant fasted-fed differences were detected for any analyte in any examined tissue (p > 0.05). No multiplicity adjustment was applied.

Plasma and tissue concentrations and *K*_*p*_ values were determined at 24 h after the fifth oral dose on Day 5 in both fasted and fed rats (n = 3 per group, Figure 7; Supplementary Table S7). In the tissue distribution study, ETN101 and M2 were quantifiable in all examined tissues, whereas M1 was below the limit of quantification (BLQ) in the brain and fat of some individuals. ETN101 showed the highest *K*_*p*_ values in the lung and kidney, M1 showed relatively high *K*_*p*_ values in the spleen, lung, and kidney, and M2 generally showed lower *K*_*p*_ values, with the largest values in the large intestine. No statistically significant fasted-fed differences in tissue concentration or *K*_*p*_ were detected for any analyte in any examined tissue (p > 0.05). Given the small group size and the number of tissues compared, the lack of a detected difference does not indicate equivalent tissue distribution.

### 3.4 Urinary and fecal excretion following intravenous injection of ETN101

**FIGURE 8:**
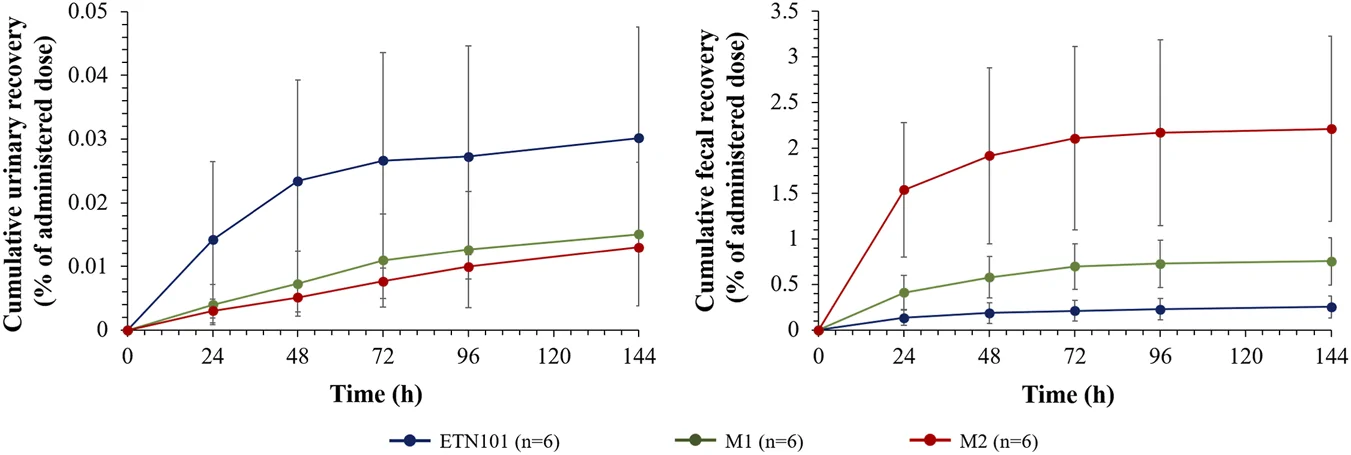
Cumulative urinary and fecal recovery of ETN101, M1, and M2 over 0–144 h after a single intravenous administration of ETN101 at 10 mg/kg in rats (n = 6, mean ± SD). Recovery of M1 and M2 is expressed on a molar-equivalent basis as a percentage of the administered ETN101 molar dose.

After intravenous administration of ETN101 at 10 mg/kg, the cumulative amounts of ETN101, M1, and M2 excreted into urine and feces were determined over 0–144 h (Figure 8; Table 6). The cumulative urinary recovery of ETN101, M1, and M2 at 144 h was approximately 0.03%, 0.02%, and 0.01% of the administered dose, respectively, and increased gradually throughout the collection period. In feces, the cumulative recovery of ETN101, M1, and M2 at 144 h was approximately 0.25%, 0.76%, and 2.21% of the administered dose, respectively. Most of the fecal excretion occurred within the first 72 h, after which the cumulative amounts changed little up to 144 h.

**TABLE 6:** Cumulative urinary and fecal recovery (% of administered dose) of ETN101, M1, and M2 over 0–144 h following a single intravenous administration of ETN101 at 10 mg/kg in rats (n = 6, mean ± SD).

Parameter	ETN101	M1	M2
Cumulative urinary recovery	0.03 ± 0.02	0.02 ± 0.01	0.01 ± 0.01
Cumulative fecal recovery	0.25 ± 0.12	0.76 ± 0.26	2.21 ± 1.02

For M1 and M2, cumulative recovery was expressed as a percentage of the administered ETN101 dose after molecular-weight correction to a molar-equivalent basis.

## 4 Discussion

### 4.1 Development and validation of an LC-MS/MS method

In this study, LC–MS/MS methods were developed and validated for the determination of ETN101 and its metabolites, M1 and M2, in rat plasma, tissues, urine, and feces. A simultaneous assay was applied to plasma, urine, and feces, while ETN101 and its metabolites in tissue homogenates were analyzed using two separate LC–MS/MS systems. A recent *in vitro* metabolism study identified a total of 34 ETN101 metabolites in human and animal hepatocytes and established that ETN101 is primarily hydrolyzed to M1 (CJM-126), which is subsequently N-acetylated to M2 by NAT1 (N-acetyltransferase 1) and NAT2 (N-acetyltransferase 2), or oxidized predominantly by CYP1A2 (Jung et al., 2024). The validated methods in the present study were applied to characterize the *in vivo* pharmacokinetic profiles, tissue distribution, and excretion of ETN101 in rats. ETN101 and its two metabolites were quantified in plasma, all examined tissues, urine, and feces to allow an integrated characterization of the ADME properties of ETN101, in which plasma reflects systemic exposure, the tissues reflect distribution, and urine and feces represent the renal and fecal excretion routes. Among these matrices, plasma, liver, urine, and feces were selected as representative matrices for the assessment of the matrix effect, extraction recovery, and process efficiency. Liver was chosen to represent the tissues as the pharmacological target organ for hepatocellular carcinoma and the principal site of ETN101 metabolism.

For each analyte, the collision energy and the other compound-dependent parameters were optimized to maximize the signal of the most abundant and selective product ion, which is the conventional approach in quantitative LC–MS/MS bioanalysis. Under these conditions, the protonated analytes of ETN101, M2, and the internal standard amlodipine were largely converted to their product ions (Figure 2), favoring assay sensitivity and selectivity over preservation of the precursor ion. A key analytical challenge was the potential for in-source fragmentation of M2 to interfere with the M1 MRM channel. The product ion of M2 (m/z 227.0) is nearly identical in mass to the protonated precursor of M1 (m/z 227.1), and in-source fragmentation of M2 was confirmed to generate a signal in the M1 MRM channel during preliminary analysis. This type of interference from structurally related metabolites has been recognized as a source of quantification error in LC–MS/MS bioanalysis (Xu et al., 2015). Therefore, complete chromatographic separation of M1 and M2 was essential for reliable quantification.

Among the six stationary phases evaluated (C18, phenyl-hexyl, C8, biphenyl, SB-Aq, and HILIC), only the Poroshell 120 SB-Aq column achieved baseline separation of M1 and M2 with acceptable peak shape and sensitivity for all three analytes. The StableBond Aqueous phase employs bidentate silane bonding chemistry that prevents stationary phase collapse under high-aqueous mobile phase conditions, thereby maintaining stable reversed-phase retention. ETN101 contains a benzothiazole moiety linked to a diaminopropanoyl group, and both M1 and M2 retain the benzothiazole aromatic core. These analytes possess both aromatic and polar functional groups, so adequate retention required a relatively high aqueous content in the mobile phase, a condition under which conventional C18 phases are susceptible to phase collapse. The SB-Aq phase provided reliable retention and specificity of M1 and M2. Acetonitrile was selected as the organic modifier over methanol, as methanol increased the retention time without improving sensitivity or resolution. Various buffer compositions were evaluated, including formic acid, acetic acid, ammonium formate, and ammonium acetate at different concentrations. A 20 mM ammonium formate buffer with 0.1% formic acid in the aqueous mobile phase provided the best selectivity and enhanced sensitivity through efficient [M + H]^+^ formation in positive ESI mode and improved peak symmetry. Gradient elution was employed to enable simultaneous determination of ETN101, M1, and M2 within a single chromatographic run with reduced analytical time, while allowing effective column washing with high organic content between injections to maintain reproducible chromatographic performance throughout the analytical batch.

For the analysis of urine and feces, the LC conditions were optimized using the same column, mobile phase composition, and gradient program as those established for plasma. However, methanol was selected for protein precipitation of urine and feces samples instead of acetonitrile, as it provided improved selectivity for ETN101, M1, and M2 in these matrices.

For the tissue samples, ETN101 and its metabolites were analyzed on two separate instruments. ETN101 carries two aliphatic amine groups and is comparatively hydrophilic, whereas M1 and M2 are more lipophilic and are retained more strongly on reversed-phase columns. Retaining and separating all three analytes under a single chromatographic condition in the complex tissue matrix was difficult, and assigning the parent drug and the metabolites to different columns and instruments gave better retention and peak shape for each. ETN101 was determined on the Agilent 6495 with the Poroshell 120 SB-Aq column, using the column and mobile phase established for plasma with a modified gradient, and M1 and M2 were determined on the SCIEX API 4000 with a Luna Omega PS C18 column, which is designed for aromatic and polar compounds and gave the best peak shape, sensitivity, and selectivity for these analytes in tissue. Running the two instruments in parallel also allowed the large number of tissue samples in the validation and distribution study to be analyzed more efficiently. On the SCIEX API 4000, 0.1% formic acid in the aqueous and organic phases with isocratic elution at 55:45 (v/v) was used for the 6 min run.

Protein precipitation was selected as the extraction method for all matrices, as it enables the high-throughput sample processing required for ADME studies generating hundreds of samples. For plasma, a single precipitation step with 0.1% formic acid in acetonitrile was sufficient. For tissues, urine, and feces, a double protein precipitation with methanol was applied to reduce matrix interference. This preparation approach gave acceptable accuracy and precision for the matrix-matched QC samples in each matrix.

Two internal standards were employed in this study: amlodipine for plasma, urine, and feces, and glipizide for tissue homogenates. During preliminary analysis of tissue samples, amlodipine exhibited a progressive increase in IS peak area with successive injections, indicating cumulative matrix effects from the complex tissue homogenate. This matrix-dependent signal drift compromised the reliability of IS normalization for tissue quantification. Therefore, an alternative IS was screened, and glipizide was selected as it demonstrated stable peak response without cumulative matrix effects throughout the analytical batch in tissue homogenates. Glipizide was applied as the IS for tissue analysis on both the Agilent 6495 and SCIEX API 4000 platforms.

The LLOQ values in plasma (2 ng/mL for ETN101 and 0.2 ng/mL for M1 and M2) were approximately 25-fold lower than those in tissues (50 ng/mL for ETN101 and 5 ng/mL for M1 and M2). This difference is attributable to the greater matrix complexity of tissue homogenates and the inherent dilution from the double protein precipitation procedure. Nevertheless, the concentrations of the analytes in most of the examined tissues at 24 h were above the LLOQ (Supplementary Table S7), indicating adequate sensitivity of the tissue method. M1 in the brain and fat was an exception, as most individual concentrations were below the LLOQ, and the corresponding values should be interpreted as approximate.

Beyond sensitivity, matrix complexity affected analyte response and recovery. ETN101 showed marked ion suppression in liver (absolute matrix effect 47%–56%) and reduced extraction recovery (63%–67%), resulting in process efficiency of 32%–35%. In feces, ETN101 showed ion enhancement (158%–160%) that partly offset reduced recovery. Substantial absolute ion suppression or enhancement was therefore observed in some matrices, particularly for ETN101 in liver and feces. Nevertheless, these effects were reproducible across the six evaluated lots, and matrix-matched calibration yielded acceptable overall accuracy and precision under the evaluated conditions. IS-normalized matrix factors, the matrix effect and recovery of the internal standards, and matrix effects in tissues other than liver were not determined, and isotope-labeled internal standards were not available, so full compliance with the ICH M10 matrix-effect assessment was not shown. Dilution integrity was not formally validated. Samples above the ULOQ were diluted with the corresponding blank matrix and gave consistent results on reanalysis, which does not substitute for a formal dilution-integrity assessment (International Council for Harmonisation, 2022).

### 4.2 Pharmacokinetic study following repeated oral administration of ETN101

Following repeated oral administration of ETN101 at 20 mg/kg once daily for 5 days, no statistically significant fasted–fed differences in AUC_0–24_ were detected for ETN101, M1, or M2 on Day 1 or Day 5. Given the small group size, these results should be regarded as exploratory rather than as a definitive assessment of a food effect (U. S. Food and Drug Administration, 2002).

Trough concentrations on Days 3–5 were consistent with attainment of steady state during once-daily dosing (Supplementary Table S6). Systemic exposure on Day 5 was similar to or modestly higher than on Day 1 without a consistent pattern across the three analytes, and the small group size does not allow accumulation to be established.

M1 showed substantially lower systemic exposure than ETN101, whereas M2 was the predominant circulating analyte, with markedly higher C_max_ and AUC_0–24_ that were comparable to or exceeded those of ETN101 in some groups. The conversion of M1 to M2 is mediated by N-acetylation through NAT1 and NAT2 (Jung et al., 2024), so the systemic exposure to both metabolites depends on N-acetyltransferase activity. This activity differs across species, and NAT2 is genetically polymorphic in humans, with distinct fast- and slow-acetylator phenotypes (Hein et al., 2000). Exposure to M1 and M2, and in particular to the predominant metabolite M2, may therefore differ between rats and humans and among individuals, and the formation and exposure of these metabolites should be confirmed in human studies.

### 4.3 Tissue distribution following repeated oral administration of ETN101

ETN101 was distributed to all examined tissues, with tissue-to-plasma concentration ratios above unity in all examined tissues and the highest ratios in the lung and kidney, indicating extensive tissue distribution. M1 showed relatively high ratios in the spleen, lung, and kidney, whereas M2 generally showed lower ratios, with the highest in the large intestine. No statistically significant fasted–fed differences were detected for any analyte in any tissue. Tissue distribution was assessed at a single 24-h time point, so the *K*_*p*_ values represent tissue-to-plasma concentration ratios at that time rather than equilibrium partition coefficients.

### 4.4 Urinary and fecal excretion following intravenous injection of ETN101

The excretion study was performed following a single intravenous administration at 10 mg/kg to evaluate excretion without the confounding effect of incomplete oral absorption. Intravenous administration ensured complete systemic availability, allowing cumulative urinary and fecal recovery to be expressed as a percentage of the administered dose. With oral administration, fecal recovery may include both unabsorbed drug and systemically absorbed drug excreted through biliary or intestinal routes, which could not be differentiated in the present study.

Urinary recovery of each analyte was ≤0.03% of the administered dose. Fecal recovery was greater than urinary recovery for each analyte but remained low in absolute terms, accounting for 0.25%, 0.76%, and 2.21% of the dose for ETN101, M1, and M2, respectively. Although M2 showed the highest fecal recovery among the three quantified analytes, it represented only 2.21% of the administered dose. The combined urinary and fecal recovery of ETN101, M1, and M2 was approximately 3.3%. These data therefore identify only minor recovery of the three measured analytes and do not establish the quantitatively dominant elimination pathway or a complete mass balance. The presence of M2 in feces is consistent with biliary and/or intestinal secretion, but the present study was not designed to distinguish these pathways. A previous *in vitro* study identified numerous additional oxidative and conjugated metabolites (Jung et al., 2024), and unmeasured metabolites, tissue retention, and other elimination pathways may account for the unrecovered fraction.

## 5 Conclusion

In the present study, simple and sensitive LC–MS/MS methods were developed and validated for ETN101, M1, and M2 in rat plasma, tissue homogenates, urine, and feces. A single assay quantified the three analytes together in plasma, urine, and feces, and two instruments were used for the tissue homogenates. After oral administration, ETN101 was slowly absorbed, and M1 and M2 were both present in plasma and declined in parallel with the parent drug. ETN101 and M2 were quantifiable in all examined tissues, whereas M1 was frequently below the LLOQ in the brain and fat. The highest ETN101 *K*_*p*_ values were in the lung and kidney. After intravenous administration, urinary recovery was negligible and the combined urinary and fecal recovery of the three analytes was approximately 3.3% of the administered dose. These methods can be selected according to the study purpose and are applicable to further preclinical pharmacokinetic and ADME studies of ETN101.

## Data Availability

The original contributions presented in the study are included in the article/Supplementary Material, further inquiries can be directed to the corresponding authors.
