# Supplementary material for: Determination of ETN101, a novel anti-hepatic cancer agent and its two metabolites in rat biological matrices using LC-MS/MS for ADME characterization
Source: Front Pharmacol. 2026 Sep 9;17:1858527. doi: 10.3389/fphar.2026.1858527 (PMC13597848; doi:10.3389/fphar.2026.1858527)
Supplement: Supplementary file 1 [file DataSheet1.pdf]

Supplementary Material

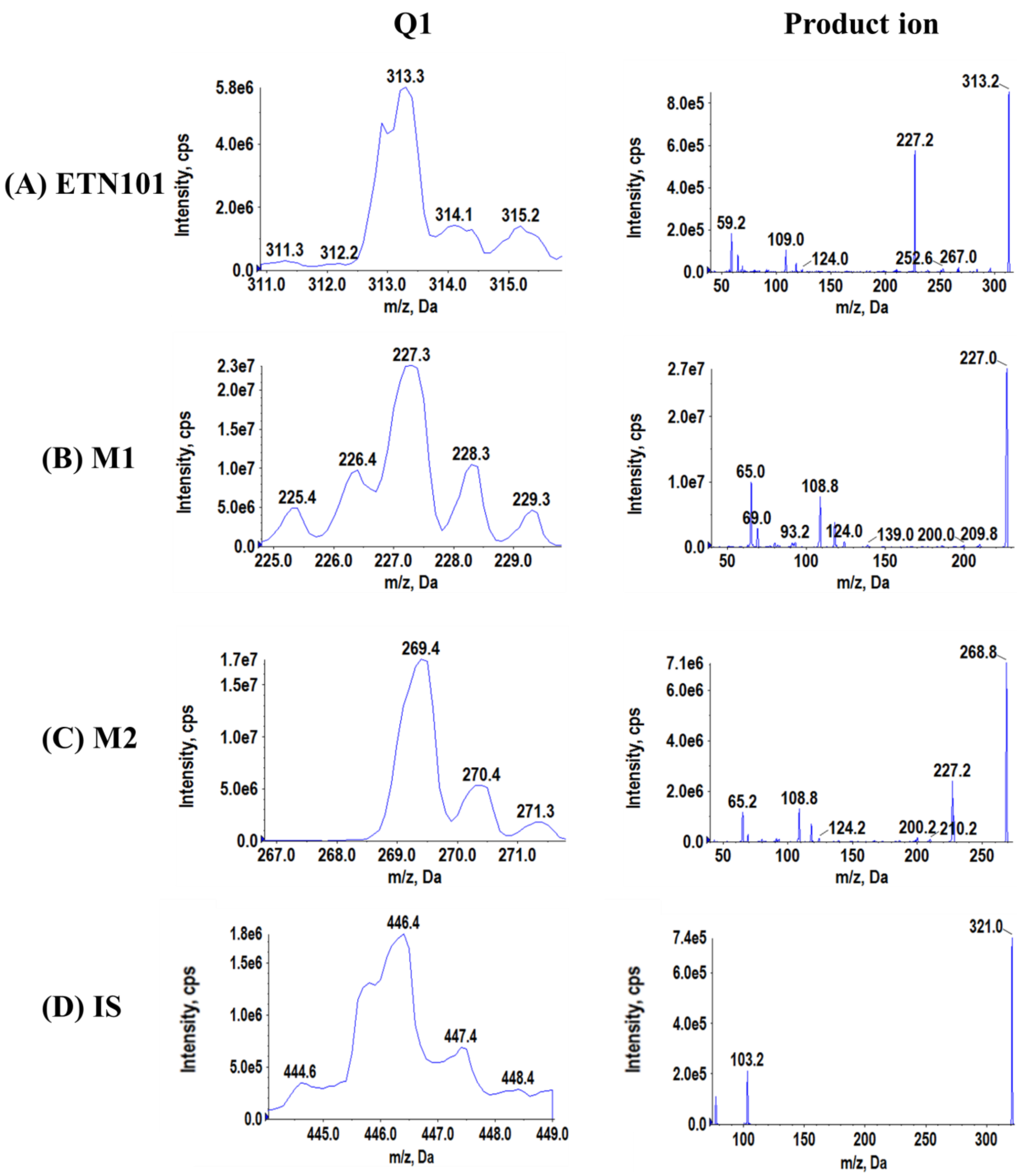

Supplementary Figure S1. Positive Q1 scan (left) and product ion mass spectra (right) of protonated (A) ETN101, (B) M1, (C) M2, and (D) glipizide (IS) obtained with the SCIEX API 4000 mass spectrometer.

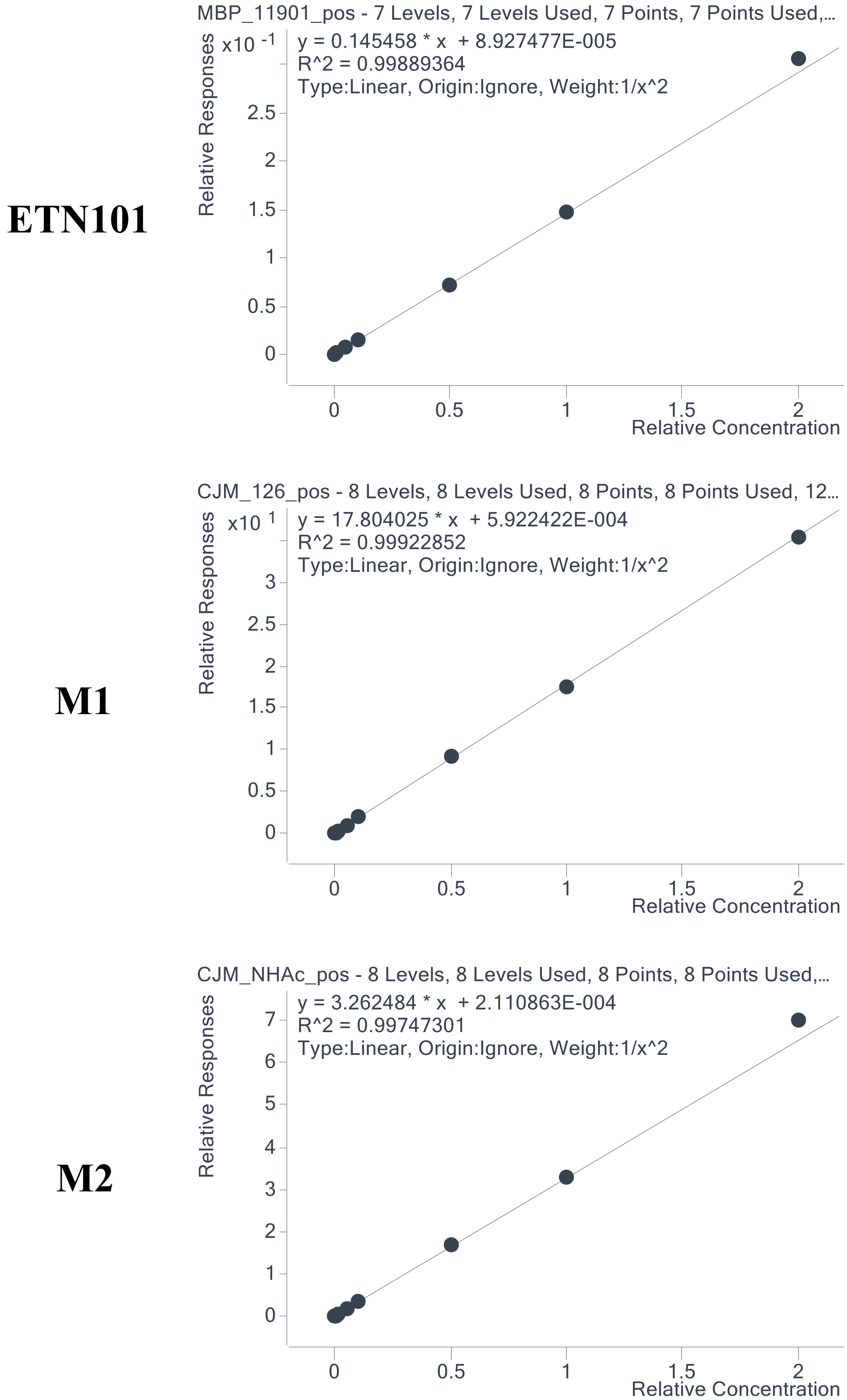

Supplementary Figure S2. Representative calibration curves for the determination of ETN101, M1, and M2 in rat plasma (r > 0.99).

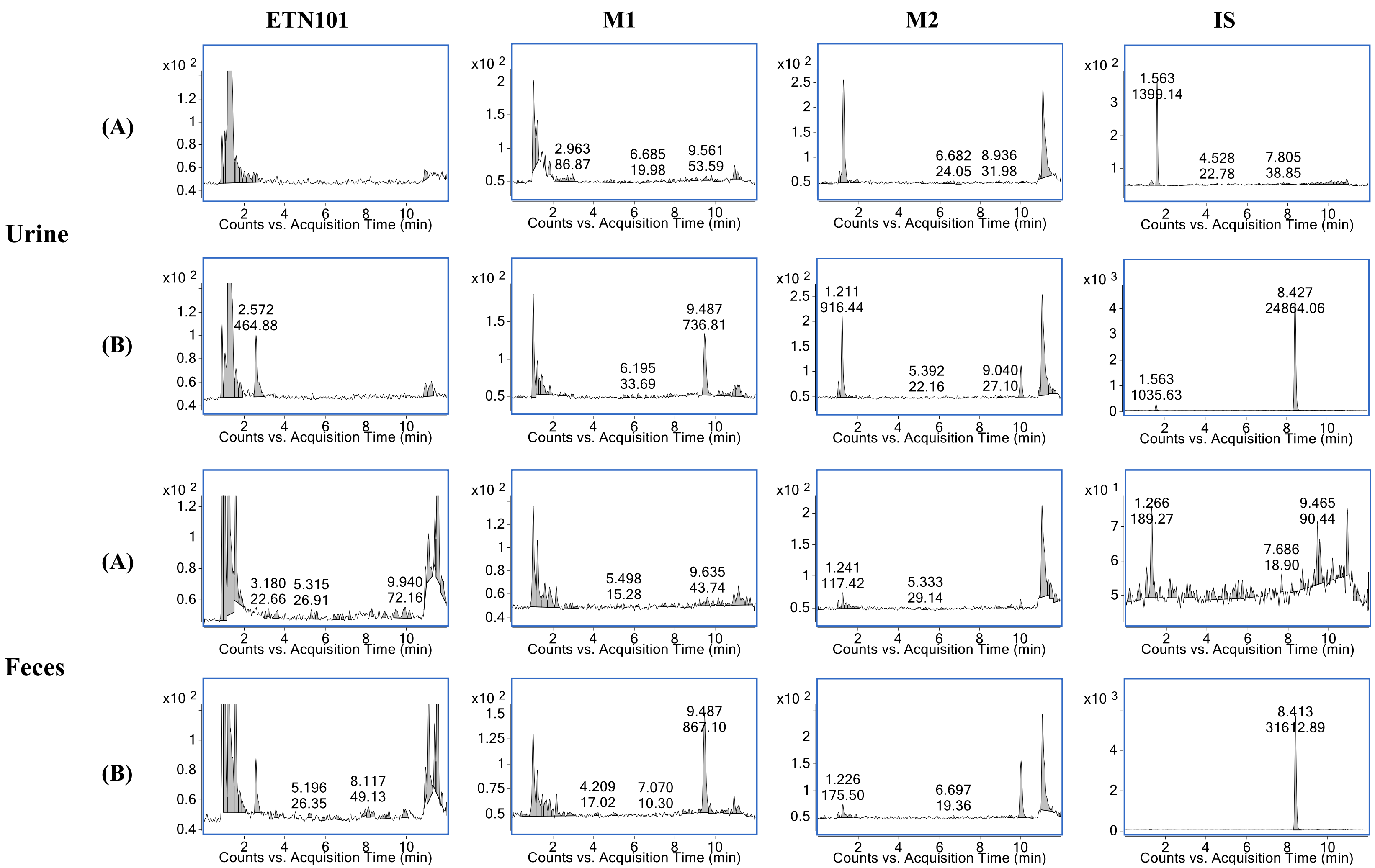

Supplementary Figure S3. Representative MRM chromatograms of ETN101, M1, and M2 in blank rat urine and fecal homogenate (left panels) and the corresponding matrices spiked at the LLOQ (right panels).

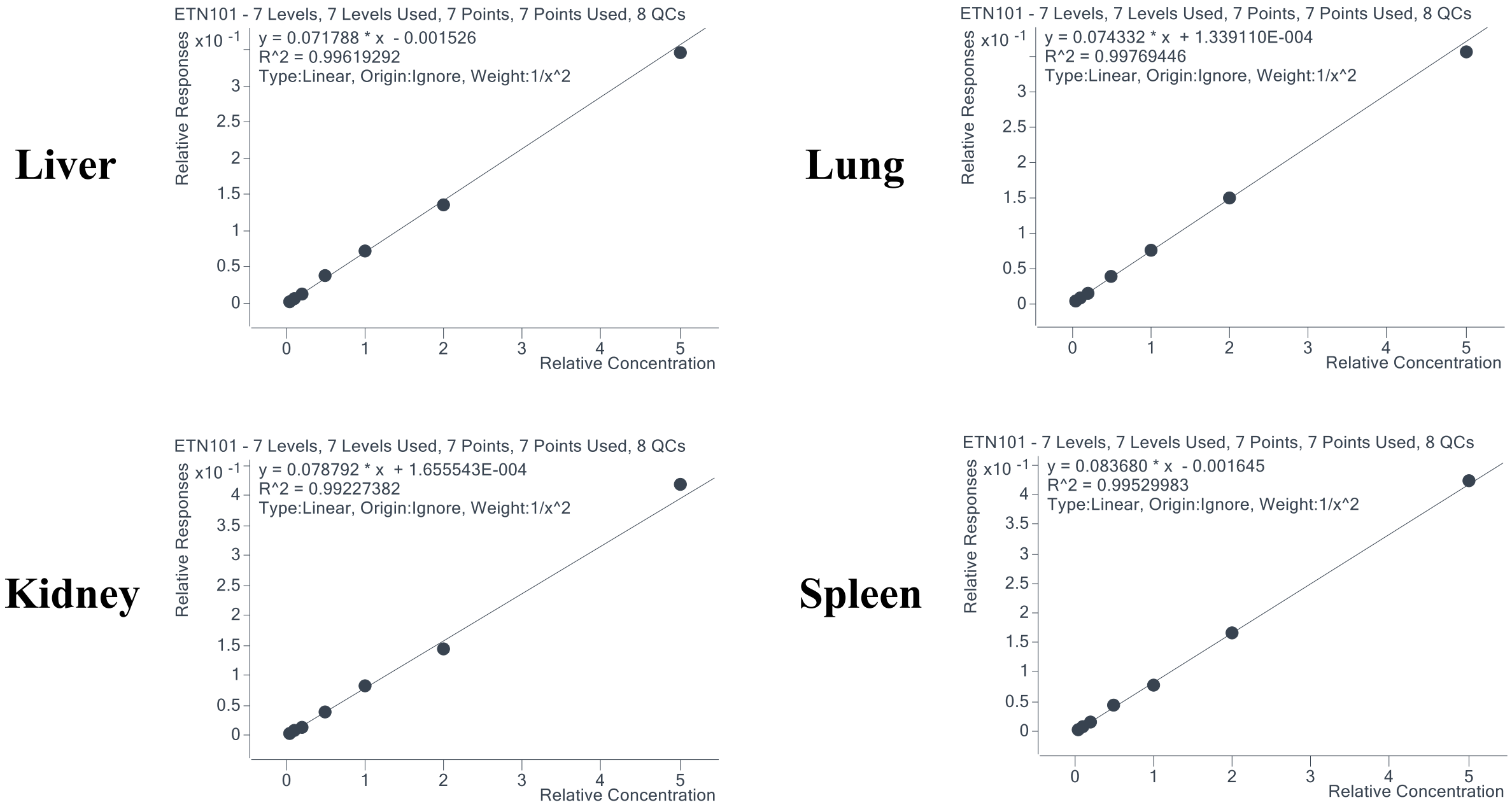

Supplementary Figure S4. Representative calibration curves for the determination of ETN101 in rat liver, kidney, lung, and spleen (r > 0.99).

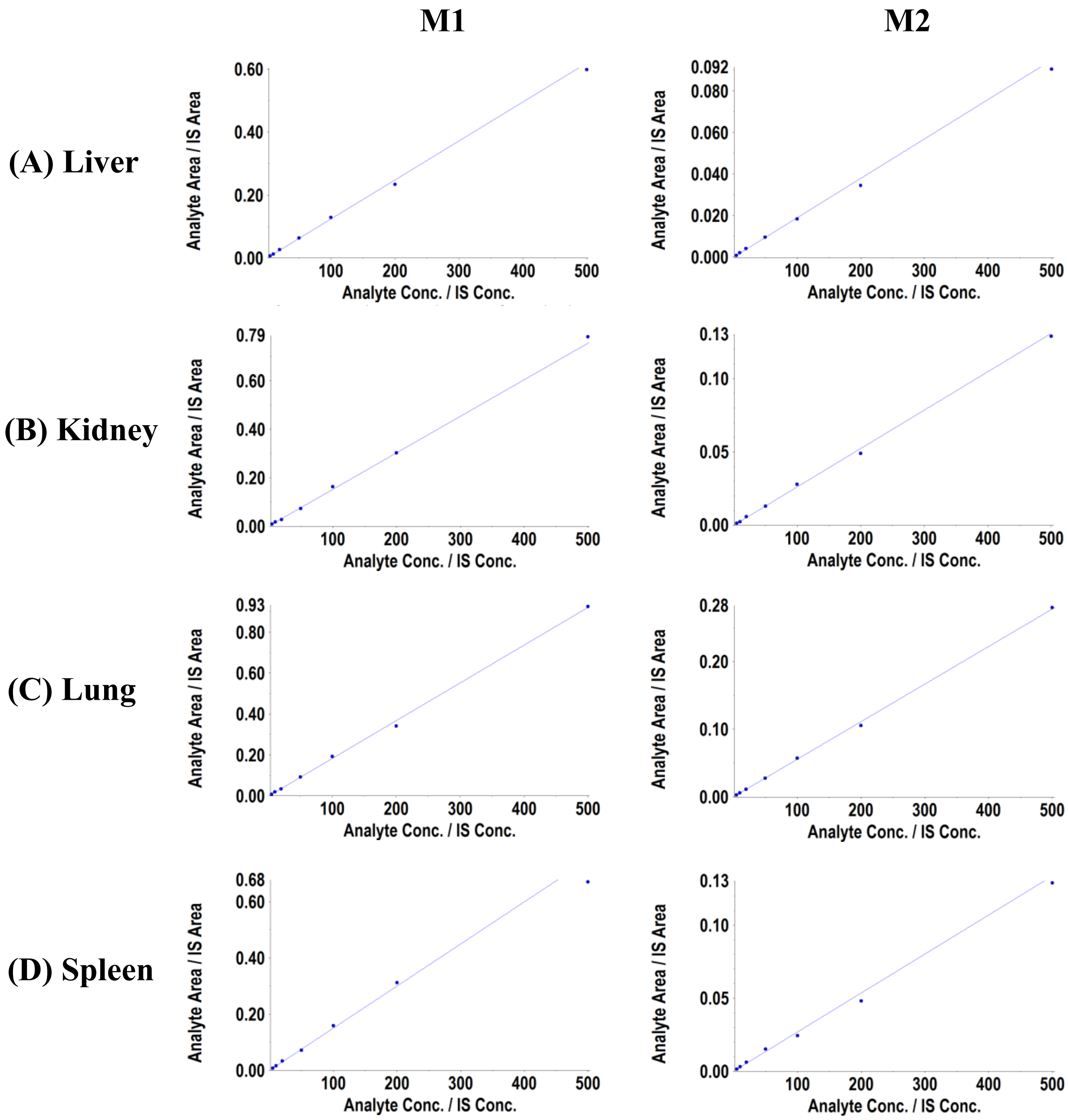

Supplementary Figure S5. Representative calibration curves for the determination of M1 (left) and M2 (right) in rat (A) liver, (B) kidney, (C) lung, and (D) spleen ($r > 0.99$).

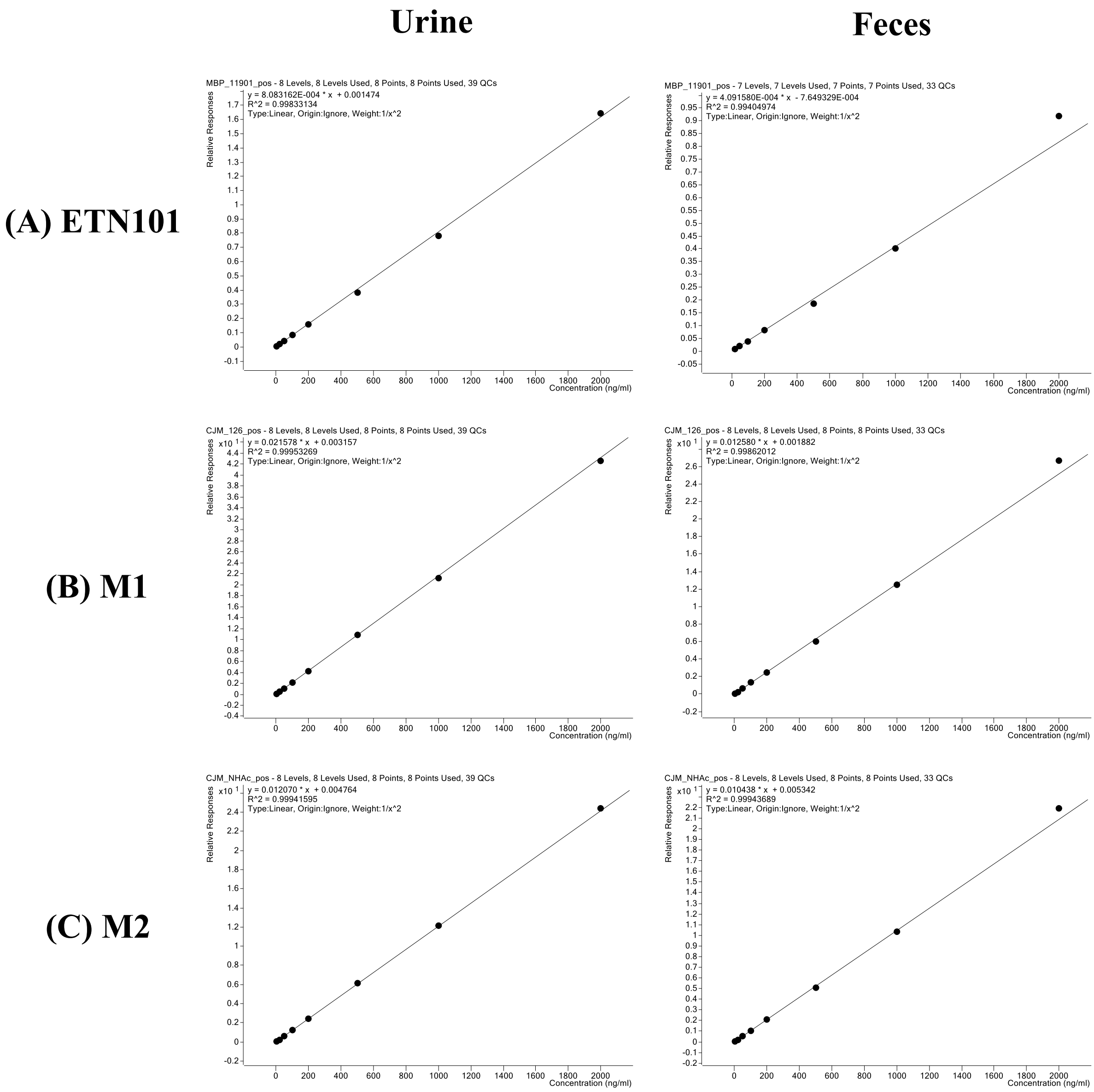

Supplementary Figure S6. Representative calibration curves for the determination of (A) ETN101, (B) M1, and (C) M2 in rat urine and feces (r > 0.99).

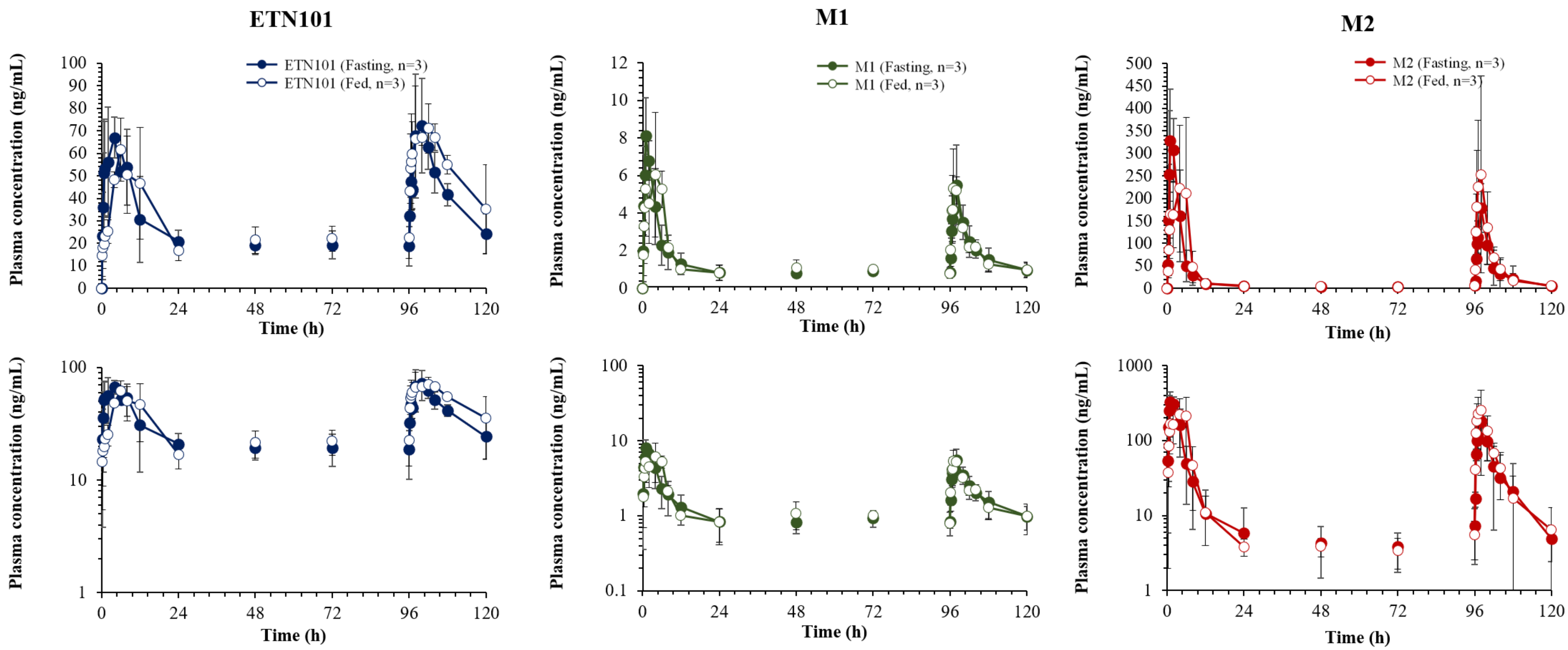

Supplementary Figure S7. Mean plasma concentration–time profiles of ETN101, M1, and M2 over the 5-day repeated-dose regimen in fasted and fed rats administered ETN101 at 20 mg/kg once daily (n = 3 per group, arithmetic mean ± SD), shown on linear and semi-logarithmic scales. Doses were administered at 0, 24, 48, 72, and 96 h; intensive profiles were obtained after Day 1 and Day 5 doses, and the 48- and 72-h points represent pre-dose samples.

Supplementary Table S1. Intra-, inter-day accuracy and precision for ETN101, M1, and M2 in rat biological matrices (n = 5)

Matrix	Concentration (ng/mL)	Intra-day (%)		Inter-day (%)	
		Accuracy	Precision	Accuracy	Precision
		ETN101			
Liver	50-4000	101.37-108.43	1.46-10.96	100.47-109.87	1.58-9.39
Lung	50-4000	89.97-109.90	1.43-4.71	90.87-108.87	0.85-6.37
Kidney	50-4000	93.87-106.60	0.64-9.18	99.13-106.37	1.13-12.73
Brain	50-4000	100.37-110.13	4.18-8.71	98.67-105.67	6.13-11.29
Heart	50-4000	99.17-105.93	3.69-6.39	96.97-108.77	2.97-10.09
Spleen	50-4000	93.33-106.97	1.01-8.75	94.73-110.20	1.11-10.52
Testis	50-4000	100.67-106.67	2.06-4.54	99.73-110.13	0.73-9.61
Thyroid	50-4000	98.73-106.90	4.59-12.65	95.20-105.93	4.87-6.90
Stomach	50-4000	98.87-107.07	1.84-5.42	98.40-111.33	2.06-8.67
Small intestine	50-4000	94.30-104.73	5.06-12.13	98.97-106.17	5.13-11.30
Large intestine	50-4000	92.17-109.80	0.54-4.33	93.47-107.93	0.52-8.55
Fat	50-4000	92.20-106.43	0.78-8.98	100.67-109.73	1.17-7.01
Muscle	50-4000	94.77-108.13	1.44-8.36	101.43-110.73	0.74-11.95
Urine	20-1600	97.30-103.47	1.85-15.00	95.80-102.67	3.51-14.74

Feces	20-1600	95.90-108.73	2.49-6.47	92.00-102.70	4.17-10.11
			M1		
Liver	5-400	88.80-109.67	1.90-4.46	96.87-109.67	2.43-4.52
Lung	5-400	99.67-110.33	1.63-7.23	103.93-107.33	0.54-11.91
Kidney	5-400	96.47-113.00	0.00-8.48	101.23-110.67	1.04-7.56
Brain	5-400	89.47-103.00	0.00-5.40	99.93-103.33	0.58-7.77
Heart	5-400	91.87-109.67	3.18-12.79	95.27-108.67	2.09-14.46
Spleen	5-400	96.33-103.20	3.59-8.16	92.20-104.57	4.95-9.72
Testis	5-400	95.70-107.33	0.65-4.78	99.43-104.03	3.99-6.67
Thyroid	5-400	107.00-109.67	1.59-4.78	96.60-103.33	2.23-9.14
Stomach	5-400	97.43-107.00	3.56-8.13	100.63-106.33	3.82-13.47
Small intestine	5-400	97.00-102.63	2.58-7.22	102.00-107.33	1.63-9.45
Large intestine	5-400	94.7-106.33	1.74-10.35	100.70-109.33	3.82-7.37
Fat	5-400	99.30-107.33	1.88-7.91	98.50-112.00	1.79-7.79
Muscle	5-400	91.27-105.33	1.32-10.89	93.30-103.90	6.58-12.68
Urine	2-1600	97.07-101.50	0.21-3.73	96.40-101.27	0.90-6.42
Feces	2-1600	91.17-105.07	1.03-3.65	94.97-100.17	0.66-8.34
			M2		

Liver	5-400	100.60-109.00	2.75-6.06	97.33-107.33	3.57-9.41
Lung	5-400	96.00-110.00	2.73-5.22	102.73-106.67	3.29-12.48
Kidney	5-400	102.27-111.33	1.87-10.36	101.03-111.00	0.90-10.31
Brain	5-400	93.43-105.60	1.08-7.32	96.60-100.83	1.25-13.53
Heart	5-400	99.10-110.00	4.55-12.73	102.53-111.00	4.77-10.64
Spleen	5-400	95.27-110.33	1.10-5.48	95.67-107.33	2.89-12.10
Testis	5-400	90.23-104.67	1.45-8.34	94.37-101.63	1.89-11.44
Thyroid	5-400	100.17-112.67	2.23-3.28	99.93-108.00	1.47-7.49
Stomach	5-400	92.80-109.33	1.40-12.37	91.27-104.77	8.86-11.82
Small intestine	5-400	97.70-107.33	1.94-4.16	98.37-108.67	1.92-9.97
Large intestine	5-400	93.93-106.30	2.13-8.94	99.00-107.33	2.19-7.13
Fat	5-400	105.33-112.33	1.85-3.01	94.87-108.77	4.88-9.14
Muscle	5-400	91.57-105.97	2.09-12.14	94.97-106.33	2.87-12.88
Urine	2-1600	100.70-110.07	3.37-5.61	98.83-104.77	0.97-13.72
Feces	2-1600	93.40-103.43	1.26-2.72	97.60-105.10	3.96-8.19

Supplementary Table S2. Stability of ETN101, M1, and M2 in rat biological matrices at low and high QC levels. Data are presented as mean ± SD (n = 3)

Matrix	Short-term (%)		Freeze-thaw (%)		Processed sample (%)		Long-term (%)	
	HQC	LQC	HQC	LQC	HQC	LQC	HQC	LQC
				ETN101				
Liver	101.20 ± 2.88	90.13 ± 2.25	97.10 ± 1.85	93.90 ± 1.78	102.00 ± 1.00	96.50 ± 3.30	91.27 ± 2.39	94.47 ± 4.47
Lung	100.00 ± 3.04	99.13 ± 0.76	105.73 ± 2.78	102.93 ± 4.37	107.30 ± 4.44	90.10 ± 3.86	104.57 ± 2.97	89.53 ± 0.93
Kidney	95.83 ± 4.92	95.83 ± 4.48	107.33 ± 1.19	94.63 ± 4.48	110.70 ± 2.35	109.30 ± 2.04	101.60 ± 14.24	94.93 ± 8.55
Brain	91.43 ± 2.31	103.63 ± 5.90	102.60 ± 7.01	95.63 ± 7.34	95.40 ± 3.60	97.57 ± 1.71	87.27 ± 1.26	101.67 ± 6.52
Heart	104.83 ± 1.02	91.43 ± 1.57	104.20 ± 0.44	90.90 ± 1.06	96.32 ± 3.93	101.30 ± 5.49	105.03 ± 1.78	100.60 ± 8.15
Spleen	96.37 ± 0.84	90.63 ± 5.52	101.87 ± 7.36	99.20 ± 2.25	90.84 ± 3.52	106.62 ± 3.53	106.67 ± 3.15	109.10 ± 3.40
Testis	92.60 ± 0.44	89.13 ± 0.78	110.07 ± 2.91	112.27 ± 2.48	88.26 ± 1.71	93.56 ± 5.58	109.27 ± 2.57	106.47 ± 2.49
Thyroid	108.17 ± 1.96	99.50 ± 4.96	107.73 ± 2.98	109.10 ± 0.96	94.77 ± 7.93	96.83 ± 5.03	90.53 ± 6.61	98.37 ± 8.83
Stomach	97.27 ± 2.74	100.80 ± 2.55	99.17 ± 10.17	87.20 ± 2.00	87.30 ± 2.34	94.50 ± 6.00	98.23 ± 1.05	102.63 ± 1.31
Small intestine	113.27 ± 1.03	106.87 ± 5.23	105.43 ± 0.78	102.03 ± 3.72	99.17 ± 1.12	90.27 ± 3.16	111.67 ± 0.81	99.43 ± 1.36
Large intestine	87.07 ± 0.75	87.53 ± 0.38	93.60 ± 1.21	92.43 ± 3.20	111.27 ± 1.62	89.10 ± 3.57	111.93 ± 3.74	104.33 ± 12.93
Fat	99.03 ± 5.24	92.20 ± 2.82	106.23 ± 3.44	107.33 ± 0.91	89.77 ± 4.28	97.20 ± 3.82	94.87 ± 4.74	99.53 ± 6.80

Muscle	102.37 ± 11.05	85.33 ± 0.32	108.97 ± 5.64	94.73 ± 6.93	99.47 ± 10.66	91.30 ± 9.63	89.57 ± 5.45	88.53 ± 2.25
Urine	96.23 ± 1.88	90.43 ± 1.97	101.07 ± 1.99	86.00 ± 0.66	96.13 ± 1.33	102.83 ± 6.57	104.80 ± 1.35	95.27 ± 1.33
Feces	85.90 ± 1.06	90.40 ± 1.91	98.77 ± 1.60	97.13 ± 1.58	101.63 ± 5.85	99.77 ± 4.86	104.63 ± 2.91	102.10 ± 9.73
M1								
Liver	87.87 ± 0.75	91.63 ± 2.15	91.30 ± 2.46	88.93 ± 5.87	100.07 ± 1.97	94.30 ± 5.49	94.40 ± 2.95	94.27 ± 4.21
Lung	96.83 ± 4.92	104.33 ± 0.58	93.17 ± 2.12	104.67 ± 3.79	108.50 ± 1.11	97.92 ± 3.02	86.80 ± 0.56	89.80 ± 3.76
Kidney	98.30 ± 1.25	105.50 ± 6.14	96.07 ± 2.89	89.80 ± 3.62	106.48 ± 1.70	103.30 ± 3.27	102.23 ± 3.22	100.43 ± 6.73
Brain	103.00 ± 0.00	99.10 ± 4.19	104.67 ± 1.53	100.67 ± 4.16	102.33 ± 1.53	101.57 ± 6.83	106.00 ± 3.00	98.60 ± 1.05
Heart	93.40 ± 6.82	108.33 ± 6.66	102.57 ± 9.31	96.40 ± 10.43	101.46 ± 1.47	88.14 ± 0.62	94.57 ± 2.87	102.43 ± 6.61
Spleen	94.93 ± 3.78	92.13 ± 3.45	94.77 ± 3.10	96.10 ± 6.60	103.48 ± 2.02	102.46 ± 3.00	95.70 ± 2.42	93.8 ± 6.16
Testis	87.93 ± 4.48	88.43 ± 2.48	86.87 ± 1.66	90.93 ± 2.25	100.48 ± 1.04	102.56 ± 2.36	97.27 ± 3.44	102.73 ± 9.34
Thyroid	86.83 ± 2.59	99.93 ± 5.10	86.67 ± 2.20	98.03 ± 4.16	93.40 ± 7.18	105.33 ± 4.16	88.10 ± 3.40	90.70 ± 2.17
Stomach	98.40 ± 3.24	110.00 ± 1.00	86.30 ± 0.56	95.37 ± 6.13	96.02 ± 0.75	105.86 ± 4.44	89.20 ± 1.87	88.73 ± 3.46
Small intestine	112.00 ± 2.00	90.80 ± 4.42	110.00 ± 3.00	100.53 ± 2.25	97.53 ± 3.27	97.63 ± 3.88	107.67 ± 2.08	104.80 ± 7.54
Large intestine	87.27 ± 1.56	87.83 ± 4.14	88.97 ± 1.39	90.00 ± 3.40	104.10 ± 2.62	109.57 ± 3.89	94.67 ± 4.48	90.03 ± 1.15
Fat	97.40 ± 1.65	103.40 ± 6.24	97.07 ± 4.53	97.77 ± 9.12	100.33 ± 5.10	102.33 ± 8.02	98.37 ± 4.15	105.17 ± 6.64
Muscle	89.87 ± 0.85	89.03 ± 1.03	95.73 ± 4.77	90.57 ± 2.39	100.60 ± 3.14	104.33 ± 3.51	100.23 ± 6.84	97.97 ± 8.78

Urine	97.47 ± 1.18	94.83 ± 3.09	99.63 ± 0.87	95.70 ± 4.06	99.13 ± 1.15	94.80 ± 2.78	98.73 ± 2.45	98.20 ± 0.62
Feces	97.40 ± 3.10	92.13 ± 4.50	103.40 ± 1.92	98.10 ± 6.77	100.17 ± 0.70	91.57 ± 5.18	98.63 ± 0.21	92.67 ± 3.19
M2								
Liver	89.50 ± 1.21	94.37 ± 6.68	95.73 ± 0.92	94.93 ± 6.63	91.93 ± 1.68	93.20 ± 4.00	100.47 ± 3.32	103.23 ± 4.90
Lung	100.50 ± 3.15	96.77 ± 3.53	105.00 ± 1.00	103.03 ± 4.54	112.70 ± 1.88	102.44 ± 5.28	97.43 ± 1.25	104.47 ± 9.39
Kidney	103.33 ± 3.21	107.00 ± 4.00	99.50 ± 1.61	103.83 ± 4.86	106.46 ± 1.44	89.04 ± 3.38	102.67 ± 1.53	97.70 ± 10.51
Brain	98.67 ± 2.06	95.13 ± 0.64	102.67 ± 1.53	95.60 ± 3.17	99.13 ± 5.58	110.33 ± 5.51	97.67 ± 2.22	108.00 ± 5.29
Heart	94.43 ± 7.41	110.33 ± 3.79	98.57 ± 7.53	99.20 ± 8.75	106.22 ± 1.72	97.76 ± 2.34	110.33 ± 4.04	109.00 ± 4.36
Spleen	102.53 ± 4.27	105.67 ± 3.21	102.90 ± 3.73	110.00 ± 1.00	106.18 ± 1.59	102.56 ± 3.43	102.00 ± 1.73	100.17 ± 9.48
Testis	105.67 ± 4.73	99.07 ± 4.47	99.87 ± 2.15	104.07 ± 8.60	103.38 ± 1.51	107.38 ± 2.64	92.87 ± 1.10	94.37 ± 4.16
Thyroid	91.57 ± 3.91	95.50 ± 10.60	97.67 ± 4.45	94.03 ± 8.95	91.00 ± 2.86	103.57 ± 7.93	89.87 ± 4.45	90.20 ± 5.37
Stomach	101.43 ± 2.89	96.70 ± 3.05	88.27 ± 1.25	96.93 ± 6.01	96.72 ± 0.65	108.02 ± 3.71	112.67 ± 3.21	100.33 ± 4.05
Small intestine	111.67 ± 4.93	91.93 ± 5.15	111.00 ± 3.46	102.67 ± 2.08	100.83 ± 7.82	105.80 ± 8.16	113.00 ± 2.00	98.97 ± 5.35
Large intestine	103.00 ± 3.00	88.50 ± 0.92	97.00 ± 0.72	94.30 ± 5.80	106.40 ± 1.25	109.73 ± 1.92	110.67 ± 4.16	99.47 ± 9.22
Fat	108.33 ± 5.51	94.17 ± 9.41	105.67 ± 5.03	98.40 ± 3.16	108.33 ± 3.51	108.33 ± 4.04	94.93 ± 4.05	106.67 ± 6.11
Muscle	110.33 ± 2.08	102.13 ± 6.34	110.67 ± 4.04	113.00 ± 1.00	101.77 ± 7.35	103.17 ± 9.31	107.33 ± 3.06	95.20 ± 10.22
Urine	100.53 ± 0.46	101.00 ± 3.75	103.90 ± 1.65	99.83 ± 1.77	105.40 ± 2.26	97.80 ± 2.23	99.50 ± 3.34	107.23 ± 4.22

Feces	92.83 ± 1.59	100.30 ± 4.68	99.33 ± 1.59	111.47 ± 2.25	101.73 ± 0.65	94.37 ± 2.32	100.47 ± 1.96	96.13 ± 3.62

Supplementary Table S3. Matrix effect of ETN101, M1, and M2 in four representative rat biological matrices (plasma, liver, urine, and feces) at LQC and HQC levels. Data are presented as mean (CV%) (n = 6)

Matrix	QC level	Matrix effect		
		ETN101	M1	M2
Plasma	HQC	94.71 (2.66%)	73.46 (4.26%)	88.70 (2.72%)
	LQC	97.93 (4.46%)	68.52 (4.88%)	83.89 (2.56%)
Liver	HQC	56.07 (14.12%)	83.79 (9.32%)	100.13 (5.29%)
	LQC	47.32 (12.28%)	75.81 (10.15%)	96.78 (4.03%)
Urine	HQC	109.27 (5.34%)	103.51 (2.35%)	111.58 (0.92%)
	LQC	117.22 (10.42%)	99.38 (2.61%)	107.55 (1.32%)
Feces	HQC	157.58 (13.85%)	102.53 (5.78%)	121.03 (8.83%)
	LQC	160.21 (14.32%)	102.48 (6.69%)	112.07 (7.11%)

Supplementary Table S4. Extraction recovery of ETN101, M1, and M2 in four representative rat biological matrices (plasma, liver, urine, and feces) at LQC and HQC levels. Data are presented as mean (CV%) (n = 6)

Matrix	QC level	Extraction recovery		
		ETN101	M1	M2
Plasma	HQC	88.14 (3.48%)	96.50 (2.18%)	94.53 (2.68%)
	LQC	93.10 (2.17%)	100.43 (2.54%)	97.41 (3.01%)
Liver	HQC	63.17 (13.11%)	92.37 (3.19%)	96.39 (1.97%)
	LQC	66.87 (13.59%)	99.13 (3.86%)	104.78 (1.39%)
Urine	HQC	85.80 (4.36%)	100.28 (2.88%)	94.41 (3.03%)
	LQC	83.19 (5.45%)	95.14 (1.75%)	89.87 (2.56%)
Feces	HQC	56.83 (10.26%)	73.27 (4.29%)	74.88 (3.73%)
	LQC	61.24 (9.68%)	80.58 (5.37%)	82.89 (2.83%)

Supplementary Table S5. Process efficiency of ETN101, M1, and M2 in four representative rat biological matrices (plasma, liver, urine, and feces) at LQC and HQC levels. Data are presented as mean (CV%) (n = 6)

Matrix	QC level	Process efficiency		
		ETN101	M1	M2
Plasma	HQC	83.57 (6.05%)	70.95 (6.15%)	83.99 (2.99%)
	LQC	91.00 (5.91%)	68.65 (6.38%)	81.69 (3.93%)
Liver	HQC	35.38 (14.95%)	77.58 (9.26%)	96.46 (4.07%)
	LQC	31.74 (10.25%)	75.24 (9.31%)	101.45 (5.03%)
Urine	HQC	93.56 (5.39%)	103.81 (2.48%)	105.44 (2.43%)
	LQC	102.14 (7.43%)	94.63 (2.97%)	96.57 (3.08%)
Feces	HQC	89.37 (10.72%)	87.07 (5.16%)	90.51 (3.31%)
	LQC	97.63 (11.04%)	82.01 (4.35%)	92.92 (4.73%)

Supplementary Table S6. Pre-dose plasma concentrations of ETN101, M1, and M2 on Days 3, 4, and 5 during once-daily oral administration of ETN101 at 20 mg/kg in fasted and fed rats (n = 3 per group, mean ± SD)

**Pre-dose day (study time)**	ETN101		M1		M2	
	Fasted	Fed	Fasted	Fed	Fasted	Fed
Day 3 (48 h)	17.87 ± 3.79	21.03 ± 4.89	0.84 ± 0.17	1.02 ± 0.38	3.35 ± 1.94	3.36 ± 1.36
Day 4 (72 h)	17.31 ± 4.4	21.65 ± 4.68	0.96 ± 0.25	1.01 ± 0.07	3.74 ± 2.33	3.01 ± 1.45
Day 5 (96 h)	16.02 ± 6.84	21.61 ± 7.63	0.97 ± 0.32	0.76 ± 0.1	6.14 ± 3.88	4.93 ± 2.66

Concentrations are expressed in ng/mL.

Supplementary Table S7. Plasma and tissue concentrations and tissue-to-plasma concentration ratios (K_p) of ETN101, M1, and M2 at 24 h after the fifth oral dose on Day 5 in fasted and fed rats administered ETN101 at 20 mg/kg once daily (n = 3 per group, mean ± SD)

Matrix	**Concentration (ng/mL or ng/g)**		**K_p**	
	Fasted	Fed	Fasted	Fed
		ETN101		
Plasma	24.27 ± 8.93	35.23 ± 19.81	-	-
Liver	1950.28 ± 128.65	2299.40 ± 979.98	86.04 ± 23.43	68.56 ± 15.23
Lung	11031.34 ± 2715.90	13983.10 ± 2533.9	481.39 ± 164.95	496.76 ± 266.68
Kidney	8107.99 ± 1350.45	9289.12 ± 4375.22	348.92 ± 60.92	275.00 ± 85.66
Brain	868.11 ± 234.23	822.82 ± 127.22	39.15 ± 19.06	27.73 ± 13.15
Heart	409.76 ± 133.10	1035.97 ± 971.46	17.83 ± 7.57	41.56 ± 46.39
Spleen	2902.83 ± 1065.32	4582.39 ± 553.33	121.79 ± 33.14	149.61 ± 52.18
Testis	1160.06 ± 219.74	1180.42 ± 198.11	53.70 ± 24.16	38.32 ± 13.56
Thyroid	1814.75 ± 791.73	969.44 ± 767.31	74.22 ± 13.25	32.76 ± 34.03
Stomach	3338.82 ± 2829.25	1276.57 ± 880.01	156.88 ± 150.09	51.58 ± 40.96
Small intestine	4459.46 ± 4238.67	2525.87 ± 2218.90	211.08 ± 221.52	103.10 ± 92.99
Large intestine	6775.21 ± 5909.92	4314.20 ± 3688.31	314.85 ± 312.69	175.11 ± 156.13
Fat	5511.07 ± 3776.79	4059.51 ± 1075.57	263.60 ± 207.78	132.95 ± 61.24
Muscle	1251.13 ± 334.90	668.51 ± 319.62	56.26 ± 26.92	20.71 ± 11.75
		M1		
Plasma	0.99 ± 0.35	0.99 ± 0.43	-	-
Liver	35.52 ± 25.37	31.31 ± 16.39	32.89 ± 13.39	31.12 ± 12.32
Lung	41.27 ± 17.20	56.60 ± 17.09	49.63 ± 38.66	59.43 ± 10.72
Kidney	36.00 ± 3.70	46.73 ± 10.37	39.07 ± 11.58	50.16 ± 10.42
Brain	4.77 ± 3.43[a]	2.92 ± 1.02[a]	4.68 ± 2.14[a]	3.02 ± 0.27[a]
Heart	19.45 ± 9.17	14.97 ± 5.76	20.24 ± 7.57	15.46 ± 4.12
Spleen	71.93 ± 48.56	72.60 ± 31.39	69.70 ± 25.71	86.37 ± 65.41
Testis	7.06 ± 3.46[a]	6.04 ± 1.85[a]	7.00 ± 2.53[a]	6.65 ± 2.90[a]

Thyroid	13.69 ± 5.37	15.41 ± 0.52	14.20 ± 3.79	17.51 ± 6.73
Stomach	7.70 ± 2.22[a]	6.39 ± 3.40[a]	8.17 ± 2.37[a]	6.66 ± 3.68[a]
Small intestine	24.17 ± 10.21	39.15 ± 25.90	28.22 ± 20.50	41.16 ± 29.84
Large intestine	15.89 ± 3.41	12.29 ± 2.65	16.89 ± 4.00	14.80 ± 8.54
Fat	4.54 ± 2.39[a]	2.93 ± 1.29[a]	4.95 ± 2.96[a]	3.67 ± 2.94[a]
Muscle	7.19 ± 3.16[a]	6.37 ± 3.24[a]	8.16 ± 5.42[a]	6.35 ± 2.32[a]
M2				
Plasma	4.92 ± 2.50	6.48 ± 6.23	-	-
Liver	43.73 ± 21.56	65.90 ± 55.33	9.41 ± 2.39	11.00 ± 1.57
Lung	27.02 ± 15.54	47.56 ± 32.35	6.41 ± 3.86	8.95 ± 3.38
Kidney	28.47 ± 12.81	40.10 ± 20.65	6.69 ± 3.40	9.77 ± 6.17
Brain	37.25 ± 27.47	46.37 ± 44.55	7.49 ± 2.61	7.36 ± 0.58
Heart	23.92 ± 16.70	30.24 ± 29.80	5.15 ± 2.31	4.32 ± 0.83
Spleen	28.10 ± 17.66	27.26 ± 13.75	6.31 ± 3.14	6.97 ± 5.05
Testis	14.75 ± 5.02	16.73 ± 6.56	3.35 ± 1.13	4.64 ± 3.71
Thyroid	18.41 ± 16.87	28.41 ± 16.65	3.96 ± 2.51	6.29 ± 3.08
Stomach	17.43 ± 11.30	20.31 ± 17.33	4.03 ± 2.34	3.43 ± 0.47
Small intestine	25.77 ± 12.15	46.13 ± 42.12	5.89 ± 2.59	8.01 ± 2.00
Large intestine	74.40 ± 44.53	90.80 ± 43.88	17.23 ± 9.56	22.10 ± 12.97
Fat	40.03 ± 44.02	31.01 ± 25.16	7.65 ± 5.34	3.17 ± 0.69
Muscle	10.87 ± 8.06	9.77 ± 4.31[a]	2.50 ± 1.57	1.16 ± 0.28[a]

Concentrations are expressed as mean ± SD in ng/mL for plasma and ng/g for tissues. K_p values are expressed as mean ± SD and are not applicable to plasma.

[a] BLQ. These values are outside the validated calibration range and are presented for descriptive purposes only; they were included in the calculation of the mean, SD, and K_p but excluded from the statistical comparison between the fasted and fed groups.
